# Genetic diversity, gene flow, and landscape resistance in a pond‐breeding amphibian in agricultural and natural forested landscapes in Norway

**DOI:** 10.1111/eva.13633

**Published:** 2023-12-20

**Authors:** Hanne Haugen, Børre K. Dervo, Kjartan Østbye, Jan Heggenes, Olivier Devineau, Arne Linløkken

**Affiliations:** ^1^ Department of Forestry and Wildlife Management Inland Norway University of Applied Sciences Hamar Norway; ^2^ Norwegian Institute for Nature Research (NINA) Oslo Norway; ^3^ Department of Biosciences Center for Ecological and Evolutionary Synthesis (CEES) University of Oslo Oslo Norway; ^4^ Department of Natural Sciences and Environmental Health University of South‐Eastern Norway University of South‐Eastern Norway Notodden Norway

**Keywords:** agriculture, forest, gene flow, genetic diversity, landscape resistance, *Triturus cristatus*

## Abstract

Genetic diversity is a key part of biodiversity, threatened by human activities that lead to loss of gene flow and reduction of effective population sizes. Gene flow is a result of both landscape connectivity and demographic processes determining the number of dispersing individuals in space and time. Thus, the effect of human impact on processes determining the level of genetic diversity must be interpreted in the context of basic ecological conditions affecting survival and recruitment. When the intensity of human impact and habitat suitability correlate, the effect on genetic diversity and gene flow may be challenging to predict. We compared genetic diversity, gene flow and landscape resistance in two contrasting landscapes in Norway for the pond‐breeding amphibian *Triturus cristatus*: a highly human‐impacted, agricultural landscape with ecologically productive habitats, and a forested landscape with less productive habitats and lower levels of human impact. Our results show that genetic diversity was higher and gene flow lower within the forested landscape. Microclimatic moisture conditions and vegetation cover were important determinants of landscape resistance to gene flow within both landscapes. There were indications that landscape resistance was increased by minor roads in the forested landscape, which was not the case for the agricultural landscape, suggesting a higher vulnerability to human interference within the landscape matrix for the populations in less productive habitats. Our findings suggest that the effect of human impact on genetic diversity may not be straightforward but modulated by the ecological conditions underlying local demographic processes. Populations within both landscapes seem to be vulnerable to loss of genetic diversity, but due to different mechanisms. This has implications for the choice of relevant management actions, that is, increasing population stability may be more relevant within an agricultural landscape still permeable for dispersal, while conserving dispersal corridors may be more appropriate in the forested landscape, to avoid isolation and increased genetic drift.

## 1 INTRODUCTION

Genetic diversity is a key factor for biodiversity, affecting adaptive potential and fitness (Hoffmann et al., 2017; Keller & Waller, 2002). However, the effect of habitat fragmentation and the creation of barriers caused by human activities have led to many species experiencing decreasing effective population sizes and loss of gene flow, and in consequence a loss of genetic diversity (Leigh et al., 2019; Rivera‐Ortíz et al., 2015). In a world increasingly impacted by humans, it is important for conservation and management to understand how modifications of natural landscapes by human infrastructure and activities affect gene flow and the spatial distribution of genetic diversity.

Gene flow is a function of the realized landscape connectivity, which can be considered an emergent property determined by landscape permeability and the number of dispersing individuals (Drake et al., 2022). The latter is related to the local habitat's ability to sustain a surplus production of individuals which may disperse to other populations (Dias, 1996). In addition to local conditions, habitat suitability is also affected by large‐scale factors, for example, climate and geology (Anderson & Ferree, 2010; Ficetola & Luigi, 2016; Shemesh et al., 2022). These large‐scale factors may give rise to landscapes where habitat suitability, and likely the total number of dispersing individuals, vary significantly on a landscape scale, potentially giving rise to considerable variation in realized connectivity.

For some species, such large‐scale ecological factors may co‐vary positively with human impact. For example, there are several pond‐breeding amphibian species that seemingly thrive in agricultural landscapes, where they often utilize artificial ponds for reproduction (Martínez‐Abraín & Galán, 2018; Valdez et al., 2021). These agricultural areas are suitable for crop production due to rich soils and suitable climatic conditions. Rich soil, often combined with agricultural runoff, may lead to nutrient‐rich ponds with high productivity and abundance of amphibian larvae (Banks & Beebee, 1988; Caballero‐Díaz et al., 2022). In otherwise colder parts of the world, a warmer climate may also be important for both crop production and the development of amphibians from egg to metamorphosis (Newman, 1998). Species that have found substitutional habitats within agricultural areas are also often still present within their original, natural habitats, such as forests (Martínez‐Abraín & Galán, 2018). In managed forests, the level of human impact is often related to the level of site productivity (Beach et al., 2005), leaving areas with nutrient‐poor or climatically harsher conditions less or unimpacted by forestry activities (Farrelly et al., 2011). Amphibians may reproduce in small and natural ponds within these areas, although the naturally low levels of plant nutrients and colder water, give less optimal conditions for reproduction and development (Burrow & Maerz, 2022; Newman, 1998), likely leading to lower production of dispersers.

If the intensity of human impacts covaries positively with habitat suitability, the end effect of human activities on landscape connectivity and the associated level of genetic diversity may be challenging to predict. Based on the observed negative effects of human activities on gene flow and effective population sizes, one may assume that the populations within landscapes with high human impact are more vulnerable to loss of genetic diversity than populations within more natural landscapes. On the other hand, if the populations within the human impacted landscapes can sustain a high production of dispersers, they may be less vulnerable and possibly experience higher gene flow, compared to populations inhabiting natural but less productive habitats. This will have major implications for management priorities.

Genetic diversity is, however, not only purely determined by the level of gene flow but also by factors affecting genetic drift, making factors like population size and the history of population fluctuations and colonization, important (Allendorf et al., 2013). In addition to the creation of barriers that reduce gene flow, human activities may affect genetic diversity through the degradation or reduction of local habitats (Lebigre et al., 2022; Schmidt et al., 2020). However, human activities may also have a positive effect through creation of substitute habitats positively affecting both population sizes and genetic parameters (Martinez‐Abrain & Jimenez, 2016). Thus, to understand the human impact on gene flow and genetic diversity, both the local habitat and the landscape matrix must be considered.

The great crested newt (*Triturus cristatus*) is a pond breeding amphibian inhabiting both natural ponds within forested areas and man‐made ponds within agricultural landscapes. The species is distributed in central and parts of northern Europe and within central areas of Asia (Wielstra & Arntzen, 2011). In many countries, it is considered either threatened or of concern (Dufresnes & Perrin, 2015) due to factors such as land cover conversion, intensive agriculture and pollution (IUCN, 2009). We studied the great crested newt in southeastern Norway with the objective to compare genetic diversity, gene flow and landscape resistance in two different landscapes representing contrasting levels of human impact and habitat productivity. We evaluated two competing hypotheses: (1) Increased human impact leads to less gene flow and lower genetic diversity and (2) Landscapes dominated by ecological suitable conditions with regard to survival or reproduction may produce a higher number of dispersers which again leads to more gene flow and possibly higher genetic diversity, compared to landscapes dominated by less productive habitats. In addition, we assessed which natural and anthropogenic landscape characteristics affect gene flow in these two study systems. Finally, we tested how genetic diversity, genetic effective population size, inbreeding, and abundance are affected by natural and anthropogenic factors within the local habitat.

## 2 MATERIALS AND METHODS

### 2.1 Study area and sampling design

**FIGURE 1:**
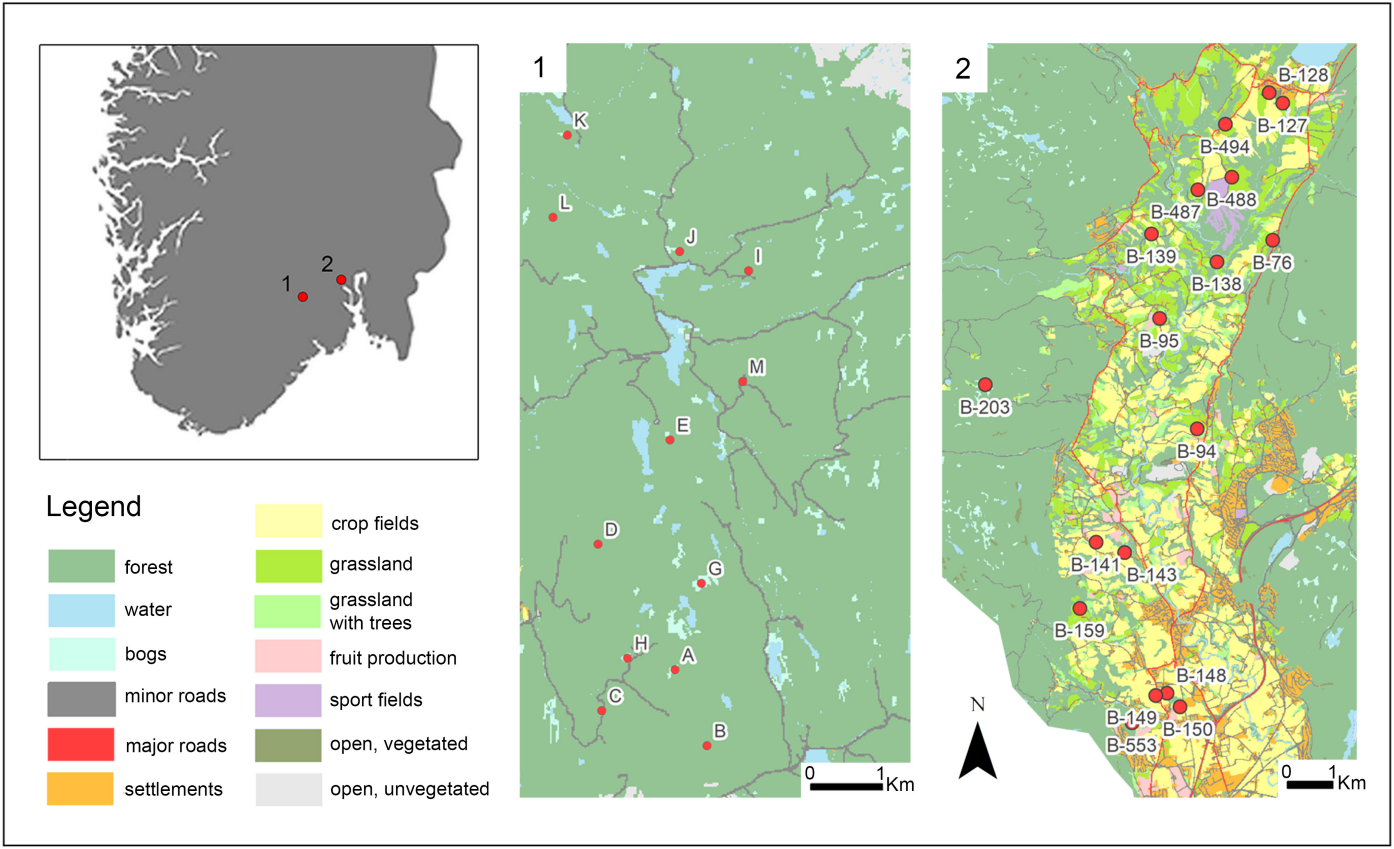
The forested landscape in Notodden (1) and the agricultural landscape in Lier (2). Red dots represent the breeding ponds of the great crested newt and letters/numbers the populations IDs. The base maps represent landcover types.

A total of 30 breeding ponds were selected from an agricultural landscape (*n* = 18) and a boreal forest landscape (*n* = 12) (Figure 1). The twelve populations in the boreal forest are located in southeastern Norway (Notodden municipality, 59°37′34.9″ N, 9°19′15.4″ E), at 242–413 m.a.s.l., with mean annual air temperature 4°C (SSV, n.d.). Average distance between ponds is 955 m (±SD = 321 m). The ponds are relatively nutrient poor (Table 1) and are located within a boreal forest dominated by pine (*Pinus sylvestris*) and spruce (*Picea abies*) on glacial moraine deposits. Approximately 60% of the area is affected by forest management activities, such as clear‐cuts and gravel roads. In the agricultural landscape, 17 breeding ponds are located directly within the agricultural area on marine clays, and one pond is in a calcareous spruce forest on the hillside west of the agricultural valley, impacted by intensive forest management (B‐203, Figure 1). The forest pond was included in the study due to its proximity to the agricultural ponds, possibly affecting the agricultural populations by contributing to gene flow. Average distance between ponds is 963 m (±SD = 593 m). The agricultural landscape is located about 50 km north‐east (Figure 1) (Lier municipality, 59°50′53.1″ N, 10°14′16.5″ E). The agricultural ponds are rich in plant nutrients (Table 1) and are located within a landscape dominated by arable fields, pastures, and some urban development, with patches for forest dominated by broad‐leaved forest and some small patches of dense spruce forests (Figure 1). The breeding ponds are located at 25 to 146 m.a.s.l, with mean annual air temperature 6.1°C (SSV, n.d.), except for the included forest pond (B‐203) at 409 m.a.s.l, with mean annual air temperature comparable to the forested study area. All breeding ponds in the agricultural part of the study system are man‐made and created in the period 1850 to 1985 (Børre Dervo, unpublished information), except for one naturally occurring pond, a small oxbow pond affected by adjacent agricultural activities (B‐138, Figure 1).

**TABLE 1:** The mean and standard deviation for breeding pond area (m^2^), elevation (m.a.s.l.), pond water chemistry (total nitrogen (Tot N (μg/L))), total phosphorous (Tot P (μg/L)), calcium content (Ca (mg/L)), within the forested (Forest) and agricultural (Agri) landscape.

Landscape	Area	m.a.s.l.	Tot N	Tot P	Ca
Forest					
Mean	1658.8	361.3	538.9	16.7	2.9
SD	899.2	50.0	210.9	13.1	1.5
Agri					
Mean	1156.9	85.4	2323.6	320.8	42.0
SD	857.3	39.4	2398.4	646.0	19.0

### 2.2 Field methods and data collection

Genetic data and catch per unit effort (CPUE) from the populations in Notodden were retrieved from a previous study (Haugen et al., 2020). In Lier, tail clips were collected (Norwegian Food Safety Authority, 27.05.2021, license #26853) during the breeding season using funnel traps in 2021 (Norwegian Environmental Agency, 09.02.2021, license #2021/1876). CPUE data were retrieved from an existing monitoring program for the period 2016–2018 (Dervo & Bærum, 2019), except for two populations (B‐127 and B‐141), which were not part of the program. For these two populations, CPUE data were derived from capture data collected during tissue sampling using 30 traps. Catch per unit effort was estimated for each pond by dividing the number of captured individuals per trap (10 traps for all populations except the two non‐monitored populations, where 30 traps were used) by the number of hours (approximately 24 h). The number of captured individuals per pond (abundance) was then estimated by multiplying with 10 traps and 24 h to convert it back to count data.

### 2.3 Genotyping

Fourteen microsatellites were used in genetic analysis (see protocol in Haugen et al. (2020)), but due to low polymorphism, one locus was removed (Tc69). A total of 894 newts were genotyped, 405 from the forested study area (mean sample size = 34, ±SD = 7), and 489 from the agricultural area (mean sample size = 27, ±SD = 8). The 13 amplified loci were tested for departure from Hardy Weinberg equilibrium in Arlequin v3.5, using 1,000,000 iterations (Excoffier & Lischer, 2010), and linkage disequilibrium using Genepop v4.7.0 and 500,000 iterations (Rousset, 2008). Significance was assessed after FDR (False Discovery Rate) correction for multiple tests (Benjamini & Hochberg, 1995). The presence of null alleles, scoring error, and large allele dropouts were tested in Micro‐Checker v2.2.3 using 10,000 iterations and α = 0.05 (Van Oosterhout et al., 2004). All loci were tested for selection neutrality in BayesScan v.2.1 using 500,000 iterations following 50,000 burn‐ins (Foll & Gaggiotti, 2008) resulting in one additional locus (Tc50) being removed from further analysis.

### 2.4 Genetic diversity, inbreeding, and effective population size

Genetic diversity was estimated as the expected heterozygosity (H_E_) and allelic richness (AR). H_E_ was calculated in Genalex v6.503 using a correction for small samples (Peakall & Smouse, 2012). Allelic richness was estimated in SpAgedi v1.5 as the mean number of alleles per loci with rarefaction (Hardy & Vekemans, 2002), a sample size of 27 individuals, and multiplying it with the number of loci to turn it into count data. As a measure of inbreeding, we included the mean internal relatedness (IR) which estimates the relatedness of each individual by the extent of shared alleles weighted by the frequency of the alleles (Amos et al., 2001). IR was estimated using GENHET v3.2 (Coulon, 2010). Effective population size (N_e_) was estimated using the linkage disequilibrium method and the software LDNE (Waples & Do, 2008). Here, we used a critical frequency threshold of 0.01 for removing low‐frequency alleles and 95% confidence intervals were calculated using jackknife. Due to negative N_e_ estimates for some populations, most likely due to high real N_e_ relative to sample size, we used the lower confidence interval instead of the estimated value. The lower confidence interval is found to be more stable than the N_e_ estimate (Macbeth et al., 2013).

### 2.5 Genetic differentiation, migration rates, and genetic structure

To assess the level of gene flow, we estimated the level of genetic differentiation, contemporary migration rates, and genetic structure within each study area separately. Genetic differentiation was estimated as pairwise *F*_ST_ and the proportion of shared alleles (Dps) (Bowcock et al., 1994). Calculation was performed in Genalex, and in R using the graph4lg package (Savary et al., 2021), respectively. Contemporary migration rates were assessed using BayesAss 3.0, which estimates the fraction of a population that has immigrated from other populations within the last two generations (Wilson & Rannala, 2003). We used 3 × 10^8^ burnins followed by 3 × 10^9^ iterations for the agricultural area, and 1 × 10^8^ burnins followed by 1 × 10^9^ iterations for the forest area. Each analysis was repeated twice to check the concordance of the results, and Tracer v1.7 was used to check for MCMC chain convergence (Rambaut et al., 2018).

Genetic population structure was analyzed using the model‐based Bayesian algorithm implemented in STRUCTURE v2.3.4 (Pritchard et al., 2000). Due to unbalanced samples in both the forested (*n* = 11–39) and the agricultural landscape (*n* = 2–31), we used two alternative methods for estimating the number of clusters (K), both methods recommended for unbalanced samples. We ran STRUCTURE using the alternative prior, initial prior = 1/maximum number of clusters (K) and the uncorrelated allele frequency model, as recommended by Wang (2017) and with the correlated allele frequency model. We ran the admixture model and a burn‐in period of 400,000 MCMC replicates followed by 1,000,000 iterations. Ten replicates were run for each K, for K = 1 to K = the number of samples (12 and 18 in the forested and agricultural landscape, respectively). Optimum K was estimated with Ln P(D) as recommended by Wang (2017) when samples are unbalanced and when using the alternative settings and the uncorrelated allele frequency model. When running STRUCTURE with the correlated allele frequency model, we used the maximum of medians estimator of K (MaxMedK), as suggested by Puechmaille (2016) for unbalanced samples. Optimum K was analyzed using STRUCTURE‐selector (Li & Liu, 2018), and replicates for optimal K was aligned using the Greedy algorithm implemented in Clumpak using 2000 iterations (Kopelman et al., 2015).

### 2.6 Comparing forest and agricultural populations

To compare expected heterozygosity (H_E_), inbreeding (IR), and effective population size (N_e_) between study areas, we used a linear regression with study area (forested vs. agricultural) as the predictor. Abundance was modeled using a negative binomial regression model due to the presence of significant overdispersion. When analyzing abundance, we included pond area as an offset to account for sampling intensity. We included only the area of the shallow parts of the pond approximated by delimiting the first six meters from the shore edge to exclude the deeper parts of larger ponds, less likely being used for breeding or egg‐laying activities (Langton et al., 2001). Allelic richness was analyzed using a Poisson regression model suitable for count data. All models were tested for spatial autocorrelation within study areas using the R‐package DHARMa (Hartig & Lohse, 2022). A significant level of spatial autocorrelation was discovered for expected heterozygosity and allelic richness. A graphical representation was made to study the distribution of genetic diversity. From this, we found that the spatial autocorrelation was likely due to a north–south gradient within the agricultural area. We accounted for this by including latitude within the regression models. The agricultural study area was the reference category in all comparisons. Beta coefficient for forested area is reported, representing the difference between the two categories.

Due to small sample sizes, we excluded three populations when analyzing allelic richness, inbreeding, and effective population size (B‐150 (*n* = 2), B‐138 (*n* = 7), G (*n* = 11)). Expected heterozygosity is more robust to small samples (Pruett & Winker, 2008), but we excluded the population with the smallest sample size (B‐150). Differences in median pairwise *F*_ST_, pairwise Dps and recent migration rates between the two study areas was assessed using a permutation test implemented in the R‐package Rcompanion and using 5000 iterations (Mangiafic, 2023).

### 2.7 Landscape effects on genetic differentiation

The effect of landscape properties on pairwise genetic differentiation was evaluated using the R package ResistanceGA (Peterman, 2018). It calculates the resistance distance between pairs of populations and optimizes the resistance surfaces based on the input genetic distance data. This is done by fitting linear mixed effects models with maximum likelihood population effects (MLPE) and using a genetic algorithm (GA) to search the parameter space (for further description of the ResistanceGA package, see Peterman (2018)). For categorical resistance surfaces, which was the only form of surfaces used here, the algorithm searches combinations of resistance values ranging between 0.001 and user‐specified max value (100). Resistance distance was calculated using Circuitscape, a software that calculates effective resistance based on circuit theory, allowing for multiple dispersal pathways (McRae et al., 2013). We used Dps as the response since this genetic distance has been found to respond faster to changes in gene flow compared to *F*_ST_ (Landguth et al., 2010).

Landscape predictors were chosen based on existing literature on the ecology of great crested newts, that is, land cover type (Hartel et al., 2010; Haugen et al., 2020; Rannap et al., 2009, 2012), moisture conditions (Dervo et al., 2016), soil pH as it is correlated with nutrient‐rich vegetation (Rydgren, 1993; Vuorio et al., 2013) and field vegetation cover (Vuorio et al., 2015).

Land cover, including forest, water (except rivers), rivers, minor roads, major roads, bogs, settlements, grassland (intensive grass production sites), cropland, grassland with trees (nonintensive grass production and pastures), open vegetated areas, open unvegetated areas, fruit production sites and sport fields, was retrieved from an existing map depicting current land cover types (NIBIO, 2019) and manually elaborated using aerial photographs from 2018 (Kartverket, 2018). Due to more recent changes in land cover types within the agricultural area, we also adjusted the land cover map in accordance with aerial photos from 2009 (Kartverket, 2009) to tentatively account for the likely presence of time lag within the genetic data (Epps & Keyghobadi, 2015).

Moisture conditions, soil pH, and field vegetation cover were modeled using LiDAR data and natural resource maps, as described in Haugen et al. (2022), but with some adjustments (described in Text S1). Moisture conditions were modeled using vegetation types as bio‐indicators for wet to very dry moisture conditions (5 levels) as response, and solar radiation load, sediment type, site index, and topographic wetness as predictors. Soil pH was modeled using vegetation types as bio‐indicators for soil calcium content (low–very high, 4 levels), and sediment type, site index, and topographic wetness as predictors. Field vegetation cover was modeled as the percentage cover of ground vegetation (height < 50 cm), and as predictors we used a lidar‐based proxy representing the amount of diffuse light reaching the forest floor and forest type (pine dominated, spruce dominated and deciduous), including an interaction between these two variables. Field vegetation cover was then categorized into three categories representing low (0%–46%), medium (46%–61%), and high (61%–100%) cover (Vuorio et al., 2015). The moisture model and the field vegetation cover model both included the effects of canopy cover. Since the canopy cover changes with time due to clear‐cuts and regrowth, we included two versions of these models representing canopy cover from year 2017 and 2008–09. In addition, we included a moisture model, which only included the effect of topography and sediment type, but not canopy cover. All maps used in the landscape resistance analysis had a resolution of 20 m.

Due to the inclusion of several predictors representing the same ecological factor, we first optimized all single resistance surfaces against the genetic distance Dps and compared the marginal *R* ^2^. Then we selected the best performing versions for each single resistance surface and included them in a multiple surface optimization procedure. Here, multiple resistance surfaces were optimized simultaneously and summed to generate new composite resistance surfaces (Peterman, 2018). We included a maximum of two single surfaces for each multiple surface optimization and compared the result with the optimized single surfaces and geographical distance. Each resistance surface was optimized twice to confirm convergence and stability of parameter estimates. Model comparisons were performed using AICc with k = the number of surfaces + intercept, that is, k = 2 for single resistance surfaces and k = 3 for composite surfaces. We also ran the “Resist. boot” function in ResistanceGA. This function utilizes a bootstrap procedure to assess the support of every optimized resistance surface included (Peterman, 2018). From this, we assessed the average AICc scores, average marginal *R* ^2^, and the proportion of bootstrap iterations where the model was selected as the top model after subsampling 75% of the samples for 10,000 iterations.

The assigned cost values for the top‐ranked predictors were retrieved. The optimization procedure was repeated three more times for the top models so that the reliability of the results could be better evaluated.

### 2.8 Local habitat at different spatial scales

The effect of pond attributes and the locale landscape around the breeding ponds on abundance, genetic diversity (H_E_, AR), effective population size (N_e_), and inbreeding (IR) was assessed using gradient boosting with component‐wise linear models implemented in the R package mboost (Hofner et al., 2014). This is a machine‐learning method that optimizes prediction accuracy and generates statistical model estimates using gradient descent techniques (Hofner et al., 2014). Stability selection was used to improve the model selection procedure by controlling for the number of falsely selected noise variables. This method has proved to perform well in high‐dimensional settings, where the number of predictors exceeds the number of samples (Hofner et al., 2015). When running the stability selection procedure, the user is required to input the assumed number of signal variables (q), which determines the number of variables selected in each subsample. Based on the limited sample size, we ran the procedure using q = 4. The procedure is robust to this assumption (Shah & Samworth, 2012). The amount of tolerated falsely selected variables (PFER) was set to 1. A spatial term was included to account for spatial variation. We assumed a gaussian distribution except for abundance and allelic richness. A negative binomial distribution was assumed suitable for the abundance data since these models tended to be overdispersed. We also included the area of the shallow parts of the pond as an offset. For allelic richness, we assumed a Poisson distribution. The four top predictors determined by the boosting procedure were included one by one in single regression models to check for statistical significance. Study area (1 and 2) was included as a fixed effect to account for the spatial structure of the study design, and the residuals were tested for spatial autocorrelation. If significant, we included a spatial correlation structure instead of study area, to account for spatial autocorrelation both within and between study areas.

Local potential habitat variables were mostly retrieved from the landscape models used in the landscape resistance analysis, but here used with a finer resolution (Table 2). In addition, we included the area of old buildings (from the 1970s or earlier) due to the possibility of them being used as overwintering sites (Dervo & van der Kooij, 2020). In addition to the calcium and plant nutrients content of the pond water, we included a variable representing the amount of sunlight reaching the pond surface (Table 2). This was estimated based on LiDAR‐data and forest type; see description in Text S1. Elevation was included to account for possible climatic effects, such as water temperature differences. Distance to nearest population was included to account for the likelihood of dispersal between ponds.

**TABLE 2:** All predictors included in the local habitat analysis, with description, abbreviation (Abbr.), and map resolution (Res.).

Predictor	Description	Abbr.	Res.
Cropland	Land used for growing cereal or vegetables	CROP	1 m
Open grassland	Areas used for intensive grass production	GRAS	1 m
Grassland with trees	Grassland with single trees or small groups of trees (i.e., non‐intensive grass production, pastures)	GTRE	1 m
Uncultivated	Uncultivated fields, with high grass or small bushes	UCLT	1 m
Unvegetated	Unvegetated areas, except major roads	UVEG	1 m
Old buildings	Buildings from the 1970s or older	BLDG	1 m
Major roads	Major roads (annual average daily traffic >1000)	ROAD	1 m
Bogs	Wetland with accumulation of peat	BOG	1 m
Forest	Areas with forest, including clear‐cuts	FOR	1 m
Pond area	The area of the pond (m^2^)	AREA	1 m
Calcium content	The amount of calcium (mg/L) in the pond water	CA	
Total nitrogen	The amount of total nitrogen (μg/L) in the pond water	TOTN	
Total phosphorus	The amount of total phosphorous (μg/L) in the pond water	TOTP	
Sun, pond surface	Amount of sunlight hitting the pond surface, estimated from solar angle, topography, and canopy cover	SUNW	1 m
High soil pH	The two highest levels of soil pH from the soil pH model	K3K4	5 m
High soil moisture	The wettest level from the soil moisture model	WET	5 m
	Low soil moisture without canopy effect	DRY	5 m
Low soil moisture	Low soil moisture with canopy effect, year 2017	DRY17	5 m
	Low soil moisture with canopy effect, year 2008–09	DRY09	5 m
	High (61%–100%) field vegetation cover, year 2017	HIGH17	16 m
Field vegetation	High (61%–100%) field vegetation cover, year 2008–09	HIGH09	16 m
	Medium (46%–61%) field vegetation cover, year 2017	MED17	16 m
	Medium (46%–61%) field vegetation cover, year 2008–09	MED09	16 m
Study area	Study areas 1 and 2 (Notodden and Lier)	LOC1	
Pond location	Forest or agricultural ponds	LOC2	
Distance to nearest population	The Euclidian distance to the nearest known population of great crested newts	DIST	
Elevation	Elevation of breeding ponds	MASL	

To explore potential effects of different spatial scales, areas with four radiuses (50, 100, 200, and 300 m) were delineated around each breeding pond to represent the potential terrestrial habitat. Inside each radius, the area of each categorized landscape property was quantified and included as predictors in the boosting procedure. All predictors at every scale were included when analyzing each response variable. Total phosphorus, total nitrogen, old buildings at 200 m scale, and unvegetated areas at 200 and 300 m scale were log‐transformed to reduce the issue of outliers.

## 3 RESULTS

No significant deviation from Hardy Weinberg equilibrium within samples was detected after correcting for multiple tests. One loci pair involving Tc50 was in linkage disequilibrium (pond M, Tcri36 – Tc50, q = 0.0065). Tc50 was also found to be under balancing selection (q = 0.001), and thus removed from further analysis.

### 3.1 Comparing forest and agricultural populations

**FIGURE 2:**
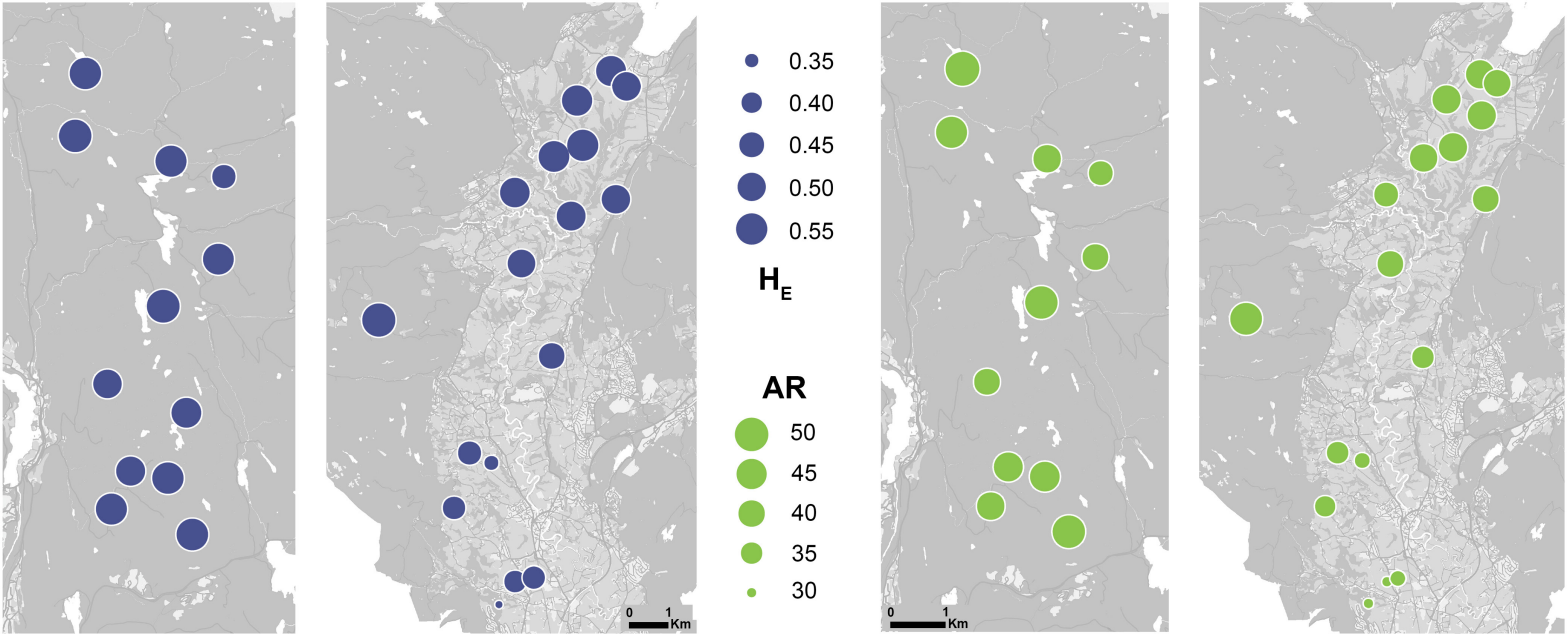
Spatial distribution of genetic diversity. Expected heterozygosity (blue, left panels) within the forested (left) landscape and agricultural (right) landscape, and allelic richness (green, right panels) within the forested (left) landscape and agricultural (right) landscape.

Genetic diversity was significantly higher within the forest landscape than in the agricultural landscape for both expected heterozygosity (Figure 2; Table S1, forest β = 0.23, SE = 0.052, *p* = 0.000014) and allelic richness (Figure 2; Table S1, forest β = 0.58, SE = 0.19, *p* = 0.0023). This contrast was aggravated if the somewhat deviating forest pond in the agricultural landscape was omitted from the analysis, resulting in increased difference in genetic diversity for both expected heterozygosity (forest β = 0.29, SE = 0.05, *p* = 0.000020) and allelic richness (forest β = 0.60, SE = 0.19, *p* = 0.0016). The effective population size, on the other hand, was not significantly different between the forest and the agricultural landscape (forest β = 2.04, SE = 12.3, *p* = 0.87), and neither was the internal relatedness (forest β = 0.0036, SE = 0.024, *p* = 0.88). Newt median abundance was, however, significantly lower within the forest landscape compared to the agricultural landscape (forest β = −1.08, SE = 0.43, *p* = 0.012).

**FIGURE 3:**
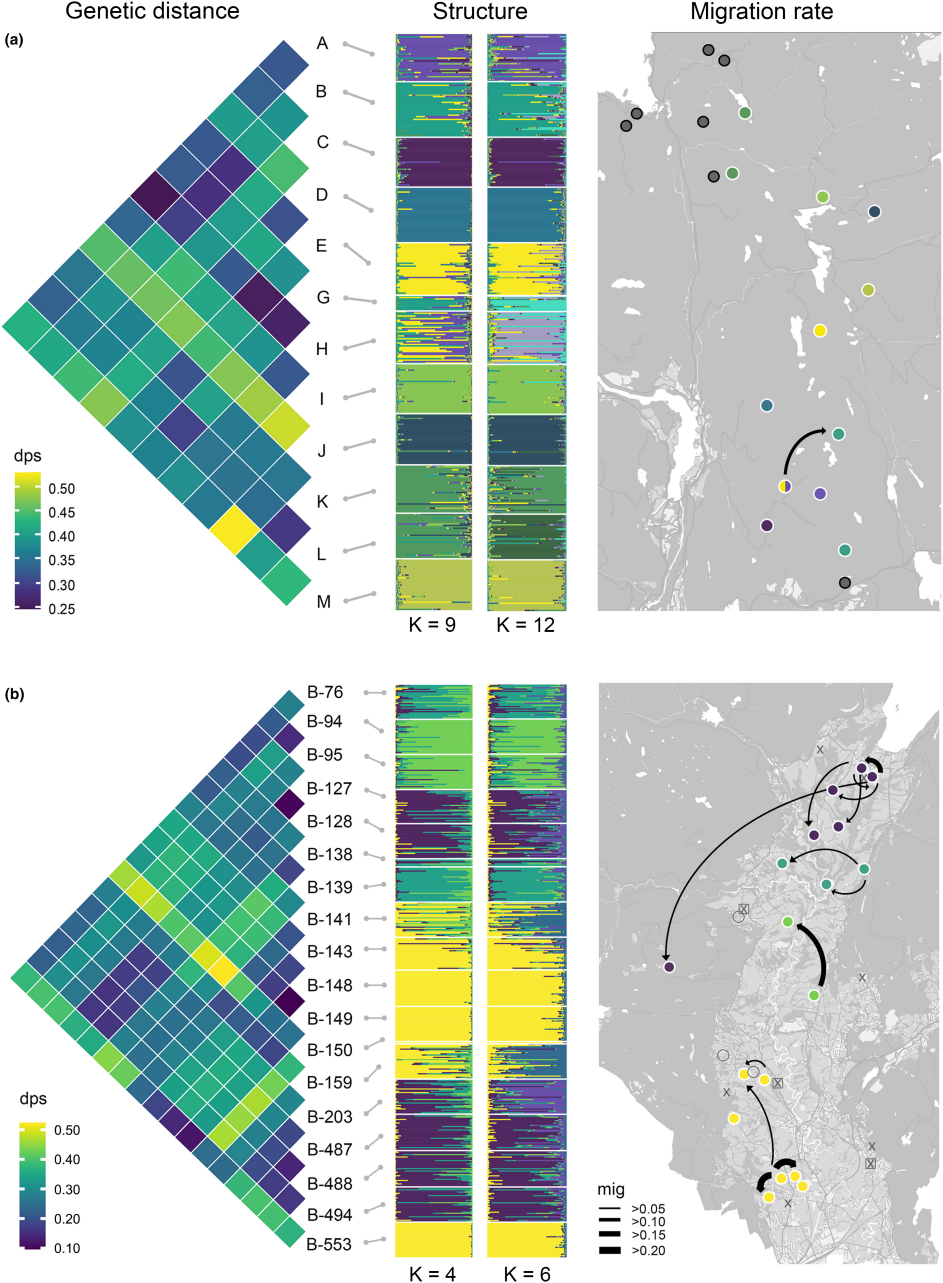
Pairwise genetic distance (Dps, left), genetic clusters (middle) from STRUCTURE, and migration rates (right) generated with Bayesass, from the forested landscape (a) and the agricultural landscape (b). Genetic clusters estimated using Ln P(D) and the uncorrelated allele frequency model (K = 4 and 9, left), and the MaxMedK method and the correlated allele frequency model (K = 6 and 12, right). Colored dots in the maps represent sampled populations and the corresponding color from the STRUCTURE analysis using Ln P(D) to estimate K. Grey dots with black rings are unsampled extant populations. Black, hollow rings represent sampled ponds with possibly undetected great crested newts. Cross signs represent historic great crested newt populations going extinct after 1990, and cross signs with black squares represent locations with ponds assumed to have been suitable for great crested newts, but that have been destroyed or degraded before 1990.

Pairwise *F*_ST_ was significantly higher in the forested landscape (Table S2, Median = 0.096, 95% CI [0.089, 0.10]) than in the agricultural landscape (Table S2, Median = 0.070, 95% CI[0.064, 0.078], *p* = 0.0004), as was pairwise Dps (Figure 3, Median = 0.387, 95% CI[0.360, 0.401] and Median = 0.287, 95% CI [0.269, 0.306], respectively, *p* = 0.00).

Median migration rate in the forest landscape (Figure 3, Median = 0.0082, 95% CI [0.0077, 0.0086]) was not significantly different from the agricultural landscape (Median = 0.0085, 95% CI [0.0080, 0.0090], *p* = 0.46). However, the highest migration rate and the highest number of relatively high migration rates (>0.10) were found in the agricultural area (Figure 3). In the forested area, there was only one incident of high migration rate (0.13), and the rest were relatively low (<0.05, Figure 3).

Both study systems showed population clustering according to the STRUCTURE results suggesting more clusters in the forested landscape (Figure 3) with the higher genetic diversity (Figure 2). Log‐likelihood values inferred by STRUCTURE provided the highest support for nine clusters in the forested landscape (Figure 3, K = 9), but only four in the agricultural landscape (Figure 3, K = 4). When using the MaxMedK‐estimator, the number of clusters were 12 in the forested landscape and 6 in the agricultural landscape (Figure 3, K = 12 and 6, respectively). In the agricultural landscape, the forest pond on the western hill became one cluster, and the southern cluster was split into two clusters. In the forested landscape, all populations were sorted into individual clusters.

### 3.2 Landscape effects on genetic differentiation

Before comparing single and composite resistance surfaces, we ran the single surface resistance optimizations procedure to determine which alternative variables representing moisture conditions, field vegetation cover, and land cover, respectively, should be included in the model comparison (Table S3). We found that the moisture model with canopy cover as it was in the years 2008–2009, performed best in both the forested and agricultural landscapes (Table S3). Field vegetation cover from 2017 performed the best in the forested landscape, while field vegetation cover from 2008 to 2009 performed better in the agricultural landscape (Table S3). The model including land cover from 2008 to 2009 performed marginally better than the one from 2017 (Table S3).

Then we compared the optimized single and composite surfaces, and geographic distance models, using AICc and two runs. For the forested landscape we found that the best model included moisture and land cover, explaining 50% of the observed variation in genetic differentiation (Table 3). The same model was also ranked third (*R* ^2^m = 46%). The second‐best model included moisture and soil pH, and explained 45% of the observed variation, but the performance varied considerably, as the same model was ranked only as number 14 for the second run (*R* ^2^m = 22%) (Table 3). The best single surface model included moisture and explained 29%–31% of the variation in genetic differentiation. Geographical distance performed relatively poorly, explaining only 10% of the observed variation (Table 3). In the bootstrap analysis, the model including moisture and land cover was still ranked first, but now the model including only moisture was ranked second. Both models were ranked as best models approximately the same proportion of bootstrap iterations (26.4%–26.5%) (Table S4).

**TABLE 3:** Models ranked by AICc, the number of cost surfaces + intercept (k), AICc, ΔAICc, and marginal *R* ^2^ for all compared landscape resistance models within the forested landscape.

Rank	Model	k	AICc	ΔAICc	R^2^m
1	Moisture + Land cover	3	−222.76	0	0.50
2	Moisture + Soil pH	3	−221.32	1.44	0.45
3	Moisture + Land cover	3	−221.03	1.73	0.46
4	Moisture	2	−219.21	3.55	0.31
5	Moisture	2	−218.82	3.94	0.29
6	Moisture + Field vegetation	3	−216.51	6.25	0.33
7	Moisture + Field vegetation	3	−216.42	6.34	0.33
8	Land cover	2	−216.29	6.47	0.16
9	Field vegetation	2	−216.29	6.47	0.23
10	Land cover	2	−216.27	6.49	0.16
11	Field vegetation	2	−216.14	6.62	0.25
12	Soil pH	2	−215.21	7.55	0.15
13	Soil pH	2	−215.12	7.64	0.17
14	Moisture + Soil pH	3	−214.79	7.97	0.22
15	Distance	2	−213.63	9.13	0.10
16	Distance	2	−213.62	9.14	0.10
17	Field vegetation + Land cover	3	−213.29	9.47	0.18
18	Field vegetation + Land cover	3	−213.29	9.48	0.18
19	Soil pH + Field vegetation	3	−213.25	9.51	0.22
20	Soil pH + Field vegetation	3	−213.25	9.51	0.22
21	Soil pH + Land cover	3	−212.13	10.63	0.16
22	Soil pH + Land cover	3	−212.13	10.63	0.16

For the agricultural landscape, the best model included land cover and soil pH, explaining 70% of the observed variation in genetic differentiation (Table 4). The same model was also ranked as second best (*R* ^2^m = 69%). The best single surface model included soil pH and was ranked 3rd (*R* ^2^m = 63%). Geographical distance performed worse than all the models including landscape properties, however, it explained quite a lot of the observed variation in genetic differentiation (58 and 59%) (Table 4). The bootstrap analysis gave similar results, that is, the model including land cover and soil pH was ranked first and second, and soil pH was ranked third (Table S5). The model including land cover and soil pH was also selected as the best model in the largest proportion of bootstrap iterations (39.8%), followed by soil pH (28.9%) (Table S5).

**TABLE 4:** Models ranked by AICc, the number of cost surfaces + intercept (k), AICc, ΔAICc, and marginal *R* ^2^ for all compared landscape resistance models within the agricultural landscape.

Rank	Model	k	AICc	ΔAICc	R^2^m
1	Land cover + Soil pH	3	−539.88	0	0.70
2	Land cover + Soil pH	3	−539.16	0.71	0.69
3	Soil pH	2	−530.61	9.27	0.63
4	Moisture	2	−528.10	11.78	0.65
5	Moisture	2	−527.81	12.07	0.65
6	Field vegetation + Soil pH	3	−525.06	14.82	0.69
7	Field vegetation + Soil pH	3	−525.04	14.84	0.69
8	Moisture + Soil pH	3	−522.10	17.78	0.61
9	Moisture + Land cover	3	−521.55	18.33	0.64
10	Moisture + Land cover	3	−521.34	18.54	0.65
11	Soil pH	2	−520.91	18.96	0.61
12	Moisture + Soil pH	3	−520.75	19.13	0.65
13	Moisture + Field vegetation	3	−513.83	26.05	0.67
14	Moisture + Field vegetation	3	−513.60	26.27	0.68
15	Land cover	2	−512.82	27.05	0.64
16	Land cover + Field vegetation	3	−510.42	29.46	0.65
17	Land cover + Field vegetation	3	−509.62	30.26	0.65
18	Field vegetation	2	−508.61	31.27	0.62
19	Field vegetation	2	−508.61	31.27	0.62
20	Land cover	2	−508.61	31.27	0.63
21	Distance	2	−508.02	31.85	0.59
22	Distance	2	−503.44	36.44	0.58

**FIGURE 4:**
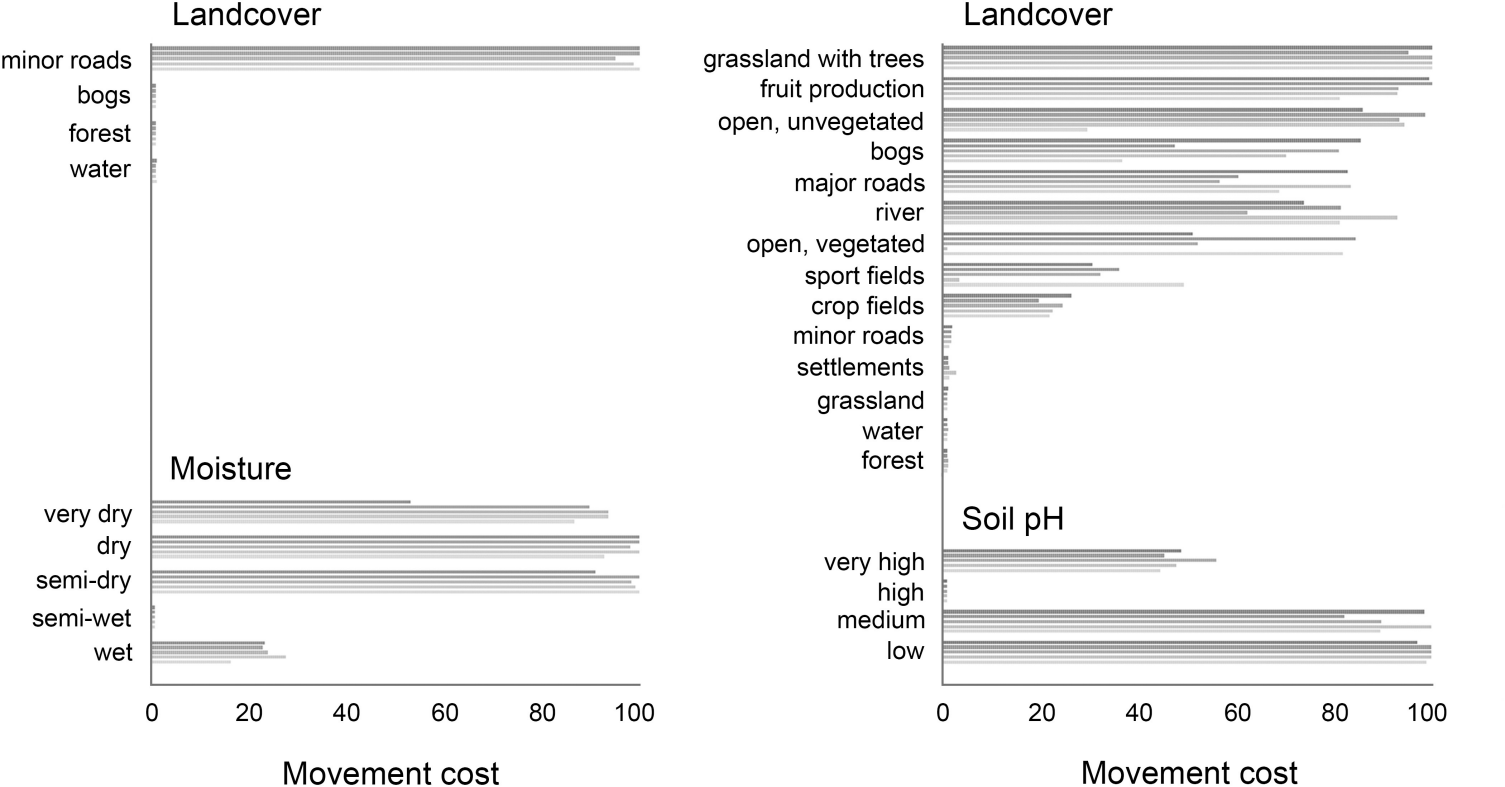
Assigned movement cost from the optimization procedure for the predictors included in the top‐ranked models for the forested (left) and agricultural (right) landscape. The bars represent the results for each run, with a total of five runs for each model.

More detailed assessment of the optimized cost values for moisture conditions in the forested landscape showed a sharp transition when moving from semiwet to semidry vegetation types, that is, semidry and drier vegetation types had high movement cost (>90), while semiwet vegetation imposed very low resistance (<2) (Figure 4). The wettest vegetation type had somewhat higher movement cost than semiwet vegetation (16–28). For land cover types within the forested landscape, forest, bogs, and water had very low movement cost (<2), while minor roads had high cost (>95) (Figure 4). In the agricultural landscape, low cost was assigned not only to minor roads but also to forest, intensive grass production sites, water (except rivers), and settlements (cost <3, Figure 4). Crop fields received relatively low movement cost (20–26). Medium to high movement cost was assigned to grassland with trees (nonintensive grass production sites and pastures), unvegetated areas, the river, bogs, major roads, and fruit production sites (>30) (Figure 4). Open, vegetated areas and sport fields were assigned varying movement costs between runs, varying from below 10 to above 45. Soil pH in the agricultural landscape received very high movement cost for low and medium soil pH (>80), and low cost for high soil pH (1) (Figure 4), while very high soil pH received a medium high cost (45–56).

### 3.3 Local habitat at different spatial scales

For all the included response variables (Table 5), genetic diversity (H_E_, AR), inbreeding (IR), effective population size (N_e_), and abundance and pooled data, the best predictor was “spatial” (Table 5), that is, the term represented by the coordinates of the breeding ponds. Effects of the other tested landscape factors were limited. Genetic diversity, represented by expected heterozygosity, was significantly negatively affected by GTRE (non‐intensive grass production sites and pastures) at the larger spatial scales of 200 and 300 m radius (Table 5, *p* = 0.015, and *p* = 0.0042, respectively). The alternative variable representing genetic diversity, allelic richness, was positively affected by MED09, that is, forest with medium field vegetation cover, at the smallest spatial scale of 50 m (Table 5, *p* = 0.046) (Table 5). In addition, effective population size was positively affected by old buildings at 50 m radius (*p* = 0.0078). The level of inbreeding (IR) was negatively affected by the amount of uncultivated vegetation at 50 and 300 m radius (*p* = 0.0016 and *p* = 0.0024, respectively). For abundance, none of the tested predictors were significantly important (Table 5).

**TABLE 5:** Results from the local habitat analysis using pooled samples from the forested and agricultural landscape with the response and the predictors.

Response	Predictor	Scale	Selection probability	β	SE	*t*‐/*z*‐value	*p*‐value
H_E_	spatial		1				
	GTRE	200 m	0.37	−0.017	0.007	−2.60	**0.015**
	GTRE	300 m	0.36	−0.025	0.008	−3.13	**0.0042**
	HIGH17	200 m	0.20	−0.0020	0.010	−0.20	0.84
	SUN		0.15	0.0038	0.009	0.43	0.67
AR	spatial		1				
	MED09	200 m	0.30	0.12	0.0006	2.00	**0.046**
	GTRE	200 m	0.21	−0.028	0.040	−0.71	0.48
	MED09	50 m	0.19	0.033	0.037	0.89	0.37
	BOG	300 m	0.18	0.053	0.031	1.71	0.087
N_e_	spatial		0.97				
	MED09	50 m	0.33	7.47	6.50	1.15	0.26
	BLDG	50 m	0.28	16.72	5.76	2.90	**0.0078**
	HIGH17	50 m	0.28	−6.97	6.12	−1.14	0.27
	GTRE	50 m	0.26	5.97	12.78	0.41	0.36
IR	spatial		0.97				
	UCLT	50 m	0.41	−0.039	0.011	−3.57	**0.0016**
	HIGH17	50 m	0.28	−0.0030	0.012	−0.25	0.81
	ROAD	100 m	0.22	0.015	0.012	1.18	0.25
	UCLT	300 m	0.22	−0.040	0.012	−3.39	**0.0024**
abundance	MED09	50 m	0.31	0.13	0.18	0.74	0.46
	HIGH17	50 m	0.26	0.10	0.18	0.58	0.57
	Log (TOTN)		0.16	0.16	0.23	0.71	0.48
	GRAS	50 m	0.15	0.28	0.20	1.35	0.18

*Note*: Regression slope coefficient β, test‐statistics (*t*‐value in all cases except for AR and abundance where *z*‐value was used) and *p*‐value are from single regression models where the predictors have been standardized. The variables are sorted by selection probability from the boosting procedure. Significant *p*‐values (<0.05) are in boldface.

## 4 DISCUSSION

Human activities may affect genetic diversity and gene flow by habitat fragmentation and creation of barriers, and by decreasing the effective population sizes. Moreover, reduction of population size and recruitment may also reduce the number of dispersers, thus negatively affecting genetic connectivity. Expected negative impacts of human activities result, however, from an interplay between type and scale of activity, model organism, local demographics, and initial natural landscape‐scale habitat suitability as determined by ecological conditions. In our study, we found that genetic diversity in a pond‐breeding amphibian was lower in an agricultural landscape, that is, more heavily impacted by humans, compared to a less impacted forest landscape. This partly supported our first and more general hypothesis, that gene flow and genetic diversity likely will decrease with increasing human impact. However, we also found indications that gene flow was lower in the forested area, suggesting that the observed lower genetic diversity in the agricultural landscape was not a consequence of loss of gene flow. Rather, lower gene flow in the forest area was more concordant with our second hypothesis, which stated that landscape‐scale ecological conditions affecting habitat suitability may be the main driver behind genetic differentiation. We found that the landscape matrix influenced gene flow within both study areas, mainly through variation in moisture levels, vegetation cover, and land cover conversion. Our findings also suggested that land cover, human‐made structures, and field vegetation cover within the local habitat, influenced genetic diversity, inbreeding, and effective population size.

### 4.1 Genetic diversity in landscapes with different levels of human impact

Although gene flow appeared to be higher, the genetic diversity was lower within the agricultural landscape compared to the forested landscape. The genetic diversity was lowest in the southern part of the agricultural area, where agricultural practices and related human impacts were more intense. In a study on the common frog (*Rana temporaria*) in Sweden, Johansson et al. (2005) found contrasting patterns of genetic diversity between areas with different levels of human impact, at different latitudes. Genetic diversity decreased with latitude, suggesting that climatic conditions was an important factor. Comparing areas within the same latitude with different levels of human impact, they found that genetic diversity was lower in the intensive agricultural landscape compared to the less intensive agricultural landscape, in the south. But this relationship was reversed in the north, where a less intensive agricultural landscape was compared with a more natural boreal forest (Johansson et al., 2005). This indicates that intensive agricultural practices may affect genetic diversity in amphibians negatively, as was also observed in a study on the marbled newt (*Triturus marmoratus*) in France (Gauffre et al., 2022). However, less intensive agricultural landscapes may be more beneficial than homogenous forest landscapes, even though the level of human impacts are lower within the latter. Johansson et al. (2005) proposed that this may be caused by the positive effect of a certain level of human impact, creating a more heterogenous habitat. We propose that the correlation between agricultural activities and suitable ecological conditions for amphibians, such as warm and nutrient rich areas, may be the important driver, not necessarily depending on the amount of human intrusion.

Both Johansson et al. (2005) and Gauffre et al. (2022) found less gene flow in the intensive agricultural landscape, suggesting that this was an important driver behind the observed lower genetic diversity. In contrast, we found that gene flow was higher within the agricultural landscape, suggesting that the lower genetic diversity here was caused by some other factors. One possibility is that since the breeding ponds were in most cases created during the 1900s, the populations in the agricultural landscape may still bear signal of the founding events (Cosentino et al., 2012; Haag et al., 2005). There could also be a higher frequency and intensity of disturbances within the agricultural landscape, caused by a range of anthropogenic influences, such as eutrophication, pollution, and land cover conversion. If these disturbances lead to temporary reductions in population size, it can lead to loss of rare alleles, that is, allelic richness (Banks et al., 2013), and if population sizes is reduced over longer time periods, it may also lead to loss of heterozygosity (Allendorf et al., 2013). This explanation is in concordance with the observation that genetic diversity was lower in the southern compared to the northern part of the agricultural landscape since the southern part was more heavily impacted by human activities.

According to our results, there were recent migration between several of the southern populations, but this did not seem to be enough to enhance genetic diversity to the same level as the northern populations. This may be because the effect of gene flow is dependent on the number of sources and the similarity of allele frequencies between the contributors and the receiver of gene flow (Biebach & Keller, 2012; Frankham et al., 2017). When looking closer at our results, we see that there were only two source populations within the southern genetic cluster, and only one population received migrants from both sources. In addition, no recent gene flow from outside the genetic cluster was detected, indicating that gene flow happened mostly between genetically similar populations. Hence, the conditions for enhancing genetic diversity within the southern genetic cluster may not have been ideal.

### 4.2 Genetic connectivity modulated by ecological context

Genetic connectivity may be modulated by the ecological context affecting population recruitment and thereby the number of potential dispersers. In Colorado, Watts et al. (2015) found higher genetic connectivity between boreal chorus frogs (*Pseudacris maculata*) populations within wetlands with longer hydroperiod duration, suggesting that the production of offspring was an important factor. In a similar study in Idaho, USA, Murphy et al. (2010) found that genetic connectivity between populations of Columbia spotted frogs (*Rana luteiventris*) was positively affected by site productivity and water temperature, and negatively affected by fish presence, that is, factors that are related to the survival and development of offspring. In our agricultural study area, the ecological conditions for production of offspring; thus, potential dispersers were likely more suitable due to warmer climate and more nutrient rich breeding ponds (Gustafson et al., 2009; Sztatecsny et al., 2004). This may explain the higher gene flow observed in this area compared to the forested study area.

An alternative explanation behind the higher gene flow in the agricultural area may be that although recruitment may be similar between study areas, a higher proportion of the newts within the agricultural populations chooses to disperse. The great crested newt seems to disperse more frequently when population density is low (Cayuela et al., 2019), as would be the case for degraded habitats. The presence of cultivated fields has been found to decrease habitat suitability for this species (Rannap et al., 2012), that is, habitat degradation could be more common in the agricultural landscape. However, we found that abundance was significantly higher within the agricultural populations, indicating that this explanation may be less relevant.

### 4.3 Microclimate, land cover, soil pH, and landscape resistance

Our study confirmed the importance of moisture conditions on amphibian dispersal (Cayuela et al., 2020). Dry areas reduced gene flow within the forested landscape. The moisture model did, however, not turn out as a highly important predictor within the agricultural landscape. This may be due to the strong effect of sediment type on the predicted moisture conditions. Areas with clay sediments, which dominated the agricultural area, were always predicted as either moist or semimoist. This was likely because the model was trained on vegetation data from inside the forests. Here, vegetation growing on clay was relatively moist because it was protected from solar radiation by a dense canopy cover. However, it seems likely that clay‐dominated areas without dense vegetation cover may experience drier microclimates.

The effect of microclimate within the agricultural parts of the landscape, however, may have been captured through the predictors land cover type and soil pH. The landscape resistance analysis suggested that land cover types representing dense vegetation (forest and heavy fertilized grassland) imposed low resistance to gene flow. Moreover, areas with no or shorter and more open vegetation (unvegetated areas, pastures, less fertilized grassland, and fruit production sites where grass is regularly mown) had a limiting effect on gene flow. Similarly, high soil pH had lower movement cost than low and medium soil pH, presumably because high soil pH is positively related to the availability of plant nutrients, which again determine the denseness of the vegetation (Weil et al., 2017). Dense vegetation provides more moisture close to the ground because it filters out more solar radiation (Stoutjesdijk & Barkman, 2014). Very high soil pH received a medium high movement cost. However, all these areas were located inside the agricultural area where anthropogenic influences probably affected the estimated cost. Grazed grassland, which was assigned a high movement cost in our study, has also been found to negatively impact newt movements in other studies. A landscape genetic study in Belgium found that grazed grassland was the most important factor affecting genetic differentiation, showing a negative effect on genetic connectivity (Cox et al., 2023). Similarly, a telemetry study in France found that great crested newts avoided open pastures when present within their local habitat (Jehle & Arntzen, 2000). The explanation may be that when grazing pressure is high, the grass vegetation is kept short, which, as mentioned, may affect the moisture conditions close to the ground.

Unexpectedly, crop fields were not assigned a very high cost to newt movement in our study. Crop fields have often been found to correlate negatively with landscape connectivity in amphibians (Covarrubias et al., 2021) but see Frei et al. (2016) and Goldberg and Waits (2010). For great crested newts crop fields entail lower habitat suitability when present within the local habitat (Rannap et al., 2012). It is, however, important to note that habitat use by adult amphibians may not necessarily be a good predictor of movement behaviors by dispersing juveniles (Rothermel & Semlitsch, 2002). Also, moving through a crop field may likely be hazardous during some seasonal periods, such as during harvest or spraying of pesticides (Brühl et al., 2013). However, there may be time periods when the fields are more permeable to movement, although this needs to be investigated further. Alternatively, if the vegetation surrounding the crop fields are suitable for newts, then they may be able to move around them. Both explanations suggest that the size of the crop fields is important, that is, smaller fields may be more easily traversed or bypassed.

Of natural features, the river seemed to limit gene flow within the agricultural landscape. Rivers have been found to be barriers for several amphibian species (Cayuela et al., 2020), including the great crested newt (Maletzky et al., 2010). Bogs were assigned a low cost in the forested study area but medium to high cost in the agricultural study area. The higher cost of bogs in the agricultural landscape was likely because bogs were concentrated in dry areas on the forested hillside, that is, correlation likely confounded the result.

### 4.4 Context‐dependent effects of transportation infrastructure

We found that minor roads had a high movement cost in the forested landscape. Roads are frequently found to impede gene flow within wild animal populations (Holderegger & Di Giulio, 2010), including amphibians (Cayuela et al., 2020). Amphibians likely experience higher mortality when crossing roads due to collisions with vehicles, and lack of vegetation cover may lead to higher risk of dehydration or predation (Cayuela et al., 2020) and avoidance behavior (Cline et al., 2016). We did not, however, find increased movement cost for the minor roads within the agricultural area. This could not be explained by the amount of traffic since the gravel roads in the forested area have generally less traffic than the minor roads in the agricultural area.

We speculate that newts may have a higher propensity for road‐crossing within the agricultural landscape. More frequent exposure to human‐altered habitats, may perhaps lead to the agricultural newts having an increased boldness due to habituation and possibly natural selection (Baxter‐Gilbert et al., 2021; Sol et al., 2013). A higher number of newts successfully crossing minor roads may also be related to the number of dispersers. The more newts that try to cross a road, the more likely it is that some will succeed. This is important because the difference between a few migrants per generation and none, or less than a few, per generation successfully crossing the road and start reproducing in the neighbor population can have major consequences for the level of genetic differentiation (Mills & Allendorf, 1996). The number of newts trying to cross the road is determined by both the number of produced dispersers within the local habitat, and the hostility of the landscape which they must pass before they reach the road. In our study, the colder and more nutrient poor breeding ponds in the forest may have given rise to less dispersers than in the agricultural area. In addition, the relatively high amount of dry vegetation types in the forested landscape may have led to a high mortality for dispersing newts, reducing the number of newts reaching the gravel roads.

### 4.5 Local habitat effects on genetic diversity, inbreeding, and effective population size

The local habitat may affect genetic parameters through its effect on population size and stability, and permeability to gene flow. In our analysis of the local habitat, we found that genetic diversity, effective population size, and inbreeding were significantly affected by the included predictors.

Expected heterozygosity was lower in areas with high amount of nonintensive grass production sites and pastures at the larger spatial scale (200‐300 m). This corroborates the results from the landscape resistance analysis showing high movement cost through this land cover type, and also an avoidance of pastures reported by Jehle and Arntzen (2000). In apparent contrast, Cox et al. (2021) found increased allelic richness with increasing number of pastures within 100 m radius from the breeding pond. This was, however, interpreted as a result of associations between pastures and hedgerows and ditches that may be utilized for habitat or dispersal corridors.

Allelic richness increased with the amount of forest with a medium field vegetation cover at the smallest scale (50 m). We had expected forest with high field vegetation cover to perform better, as it was found to be preferred by great crested newts in a study in Finland (Vuorio et al., 2015). However, the forest with high vegetation cover in our study area was correlated with the dry vegetation types; thus, medium field vegetation cover was probably to be preferred due to somewhat moister microclimate.

We found that old buildings within 50 m had a positive effect on effective population size. Old buildings can have cracks and crevices that may be utilized for overwintering (Dervo & van der Kooij, 2020). If the number of natural hibernation sites are a limiting factor, then the presence of artificial structures such as buildings may play an important role in maintaining high and stable population sizes. A study on the great crested newt close to the agricultural study area found that newly created artificial hibernacula was utilized relatively soon after creation, indicating a possible limitation of suitable overwintering sites (Dervo et al., 2018).

Inbreeding was significantly lower if there were more uncultivated fields within 50 m and 300 m radius of the breeding pond. These areas may represent good terrestrial habitat, with dense vegetation protecting against solar drying and predation. This is in accordance with a Danish study which found that the width of the uncultivated sector around the pond had a positive effect on presence of great crested newts (Rannap et al., 2009).

### 4.6 Some caveats when interpreting genetic differentiation and gene flow

We used genetic differentiation as proxy for gene flow; however, genetic differentiation is also affected by the effective population size as it affects the intensity of genetic drift (Prunier et al., 2017). Thus, if effective population sizes in the agricultural area had been generally higher than in the forested populations, then this could have confounded the results. However, we found that effective population size did not differ significantly between the landscapes, giving more support to the interpretation of actual higher gene flow within the agricultural area.

Another issue is the presence of unsampled populations intervening between sampled populations, which can cause overestimation of migration rates (Beerli, 2004). All small ponds within vicinity of the sampled breeding ponds have been monitored at least once. This makes it less likely that we have had unsampled populations intervening between sampled populations. There is, however, the possibility that some populations have been overlooked due to very low densities of newts. These are less probable to have contributed much to the gene flow due to likely low production of dispersers. The extinct populations on the other hand we know little about, and some may have contributed to gene flow before they disappeared. There are a few populations that went extinct after 1990s, that may have contributed to recent gene flow (Figure 3, cross‐symbols). However, this issue does not change the conclusion that gene flow was higher within the agricultural landscape compared to the forested landscape. Rather, it means that the observed pattern may be affected by time lag and are partly representing the population distribution from recent past. The landscape from the recent past is also an agricultural landscape, relatively similar to the current landscape when comparing it with aerial photos from the 1970s. The agricultural practices, however, may have changed over time; thus, our findings may not completely mirror the current level of human impact.

### 4.7 Implications for management

Human activities are an important driver behind loss of genetic diversity within wild animal populations (Leigh et al., 2019; Schmidt et al., 2020). However, the effect of such activities may differ with the landscape‐scale ecological conditions affecting the production of dispersers. This can entail different management efforts and perspectives relative to the different landscape contexts.

We found that both the agricultural populations and the forest populations are vulnerable to loss of genetic diversity, but due to different mechanisms. The forest populations seem to have low levels of gene flow, and even small barriers such as minor roads may increase genetic differentiation. If these populations become isolated, then genetic drift will likely lead to a loss of genetic diversity (Lande, 1995). Thus, management should focus on maintaining suitable conditions for movement within the landscape between breeding ponds, that is, avoid activities leading to drier microclimate, permanent removal of vegetation cover, and other kinds of barriers to movement. Forestry, and associated roadbuilding, is the main human impact within the forested landscape. Timber removal have shown to have a negative effect on abundance for a range of salamander species, likely due to higher solar radiation load and increased energy requirements, clearcutting being more negative than partial harvest (Tilghman et al., 2012). Thus, to maintain gene flow between populations, the intensity and extent of forestry activities in the landscape between populations should be considered with caution.

The agricultural populations seem to have a relatively high amount of gene flow, but still genetic diversity was lower than in the more natural populations. This was likely due to a combination of more fluctuating population sizes and relatively recent founding events. Loss of genetic diversity may not be easily restored by gene flow, if gene flow is coming from few and genetically similar populations (Biebach & Keller, 2012; Frankham et al., 2017). However, genetic diversity could be enhanced by increasing connectivity from populations less similar with regards to allele frequencies, that is, introduce gene flow from populations that are not currently connected. Such actions, however, must be considered carefully with regard to spread of diseases and pests, and loss of local adaption (Frankham, 2015; Frankham et al., 2011). An important focus should be to avoid loss of genetic diversity in the first place. This would entail maintaining suitable habitat area and quality, such as uncultivated fields and forest with sufficient field vegetation cover and reduce disturbances. Overwintering sites are also important, and the removal or restoration of old buildings may affect survival negatively. Maintaining suitable conditions for reproduction and survival would also likely benefit genetic connectivity.

## 5 CONCLUSION

Human activities may impact genetic diversity and gene flow within both productive and less productive habitats, but the drivers behind this impact may vary. We found that populations within more natural but less productive habitats were more vulnerable to loss of gene flow by relatively minor human impacts within the landscape matrix. On the other hand, populations within more productive habitats, seemed to maintain higher levels of gene flow despite high human impact. However, the productive populations had lower genetic diversity, likely due to more disturbances reducing population sizes, and more recent founding events. Different drivers behind the loss of genetic diversity entails different management priorities. Maintaining population stability to avoid genetic bottlenecks, may be a conservation priority for the populations within agricultural areas, provided the landscape is still permeable for movement. Conservation management should increase the amount of area with uncultivated fields or forest with sufficient field vegetation cover in the local areas around breeding ponds and provide sufficient overwintering sites. Conserving dispersal corridors, to avoid isolation and increased genetic drift, may be more important in the forested landscape.

## CONFLICT OF INTEREST STATEMENT

The authors declare no conflict of interest.

## BENEFIT‐SHARING STATEMENT

Benefits from this research accrue from the sharing of our data and results on public databases as described above.

## Supporting information


Tables S1–S5.

Text S1.

## Data Availability

Data for this study are available in Dryad at https://doi.org/10.5061/dryad.7d7wm3825.

## References

[eva13633-bib-0001] Allendorf, F. W. , Luikart, G. , & Aitken, S. N. (2013). Conservation and the genetics of populations (2nd ed.). Wiley‐Blackwell.

[eva13633-bib-0002] Amos, W. , Worthington Wilmer, J. , Fullard, K. , Burg, T. M. , Croxall, J. P. , Bloch, D. , & Coulson, T. (2001). The influence of parental relatedness on reproductive success. Proceedings of the Royal Society B: Biological Sciences, 268(1480), 2021–2027. 10.1098/rspb.2001.1751 PMC1088844 11571049

[eva13633-bib-0003] Anderson, M. G. , & Ferree, C. E. (2010). Conserving the stage: Climate change and the geophysical underpinnings of species diversity. PLoS One, 5(7), e11554. 10.1371/journal.pone.0011554 20644646 PMC2904386

[eva13633-bib-0004] Banks, B. , & Beebee, T. J. C. (1988). Reproductive success of Natterjack toads Bufo calamita in two contrasting habitats. The Journal of Animal Ecology, 57(2), 475–492. 10.2307/4919

[eva13633-bib-0005] Banks, S. C. , Cary, G. J. , Smith, A. L. , Davies, I. D. , Driscoll, D. A. , Gill, A. M. , Lindenmayer, D. B. , & Peakall, R. (2013). How does ecological disturbance influence genetic diversity? Trends in Ecology & Evolution, 28(11), 670–679. 10.1016/j.tree.2013.08.005 24054910

[eva13633-bib-0006] Baxter‐Gilbert, J. , Riley, J. L. , & Measey, J. (2021). Fortune favors the bold toad: Urban‐derived behavioral traits may provide advantages for invasive amphibian populations. Behavioral Ecology and Sociobiology, 75(9), 1–13. 10.1007/s00265-021-03061-w

[eva13633-bib-0007] Beach, R. H. , Pattanayak, S. K. , Yang, J.‐C. , Murray, B. C. , & Abt, R. C. (2005). Econometric studies of non‐industrial private forest management: A review and synthesis. Forest Policy and Economics, 7(3), 261–281. 10.1016/S1389-9341(03)00065-0

[eva13633-bib-0008] Beerli, P. (2004). Effect of unsampled populations on the estimation of population sizes and migration rates between sampled populations. Molecular Ecology, 13(4), 827–836. 10.1111/j.1365-294X.2004.02101.x 15012758

[eva13633-bib-0009] Benjamini, Y. , & Hochberg, Y. (1995). Controlling the false discovery rate: A practical and powerful approach to multiple testing. Journal of the Royal Statistical Society: Series B: Methodological, 57(1), 289–300. 10.1111/j.2517-6161.1995.tb02031.x

[eva13633-bib-0010] Biebach, I. , & Keller, L. F. (2012). Genetic variation depends more on admixture than number of founders in reintroduced alpine ibex populations. Biological Conservation, 147(1), 197–203. 10.1016/j.biocon.2011.12.034

[eva13633-bib-0011] Bowcock, A. M. , Ruiz‐Linares, A. , Tomfohrde, J. , Minch, E. , Kidd, J. R. , & Cavalli‐Sforza, L. L. (1994). High resolution of human evolutionary trees with polymorphic microsatellites. Nature, 368, 455–457. 7510853 10.1038/368455a0

[eva13633-bib-0012] Brühl, C. A. , Schmidt, T. , Pieper, S. , & Alscher, A. (2013). Terrestrial pesticide exposure of amphibians: An underestimated cause of global decline? Scientific Reports, 3(1), 1135. 10.1038/srep01135 23350038 PMC3553602

[eva13633-bib-0013] Burrow, A. , & Maerz, J. (2022). How plants affect amphibian populations. Biological Reviews of the Cambridge Philosophical Society, 97(5), 1749–1767. 10.1111/brv.12861 35441800

[eva13633-bib-0014] Caballero‐Díaz, C. , Sánchez‐Montes, G. , Gómez, I. , Díaz‐Zúñiga, A. , & Martínez‐Solano, Í. (2022). Artificial water bodies as amphibian breeding sites: The case of the common midwife toad (*Alytes obstetricans*) in Central Spain. Amphibia‐Reptilia, 43, 395–406.

[eva13633-bib-0015] Cayuela, H. , Schmidt, B. R. , Weinbach, A. , Besnard, A. , Joly, P. , & Vander Wal, E. (2019). Multiple density‐dependent processes shape the dynamics of a spatially structured amphibian population. The Journal of Animal Ecology, 88(1), 164–177. 10.1111/1365-2656.12906 30280381

[eva13633-bib-0016] Cayuela, H. , Valenzuela‐Sánchez, A. , Teulier, L. , Martínez‐Solano, Í. , Léna, J.‐P. , Merilä, J. , Muths, E. , Shine, R. , Quay, L. , Denoël, M. , Clobert, J. , & Schmidt, B. R. (2020). Determinants and consequences of dispersal in vertebrates with complex life cycles: A review of pond‐breeding amphibians (Vol. 95, pp. 1–36). The University of Chicago Press.

[eva13633-bib-0017] Cline, B. B. , Hunter, M. L. , & Parmenter, R. R. (2016). Movement in the matrix: Substrates and distance‐to‐forest edge affect postmetamorphic movements of a forest amphibian. Ecosphere, 7(2), e01202. 10.1002/ecs2.1202

[eva13633-bib-0018] Cosentino, B. J. , Phillips, C. A. , Schooley, R. L. , Lowe, W. H. , & Douglas, M. R. (2012). Linking extinction‐colonization dynamics to genetic structure in a salamander metapopulation. Proceedings of the Royal Society B: Biological Sciences, 279(1733), 1575–1582. 10.1098/rspb.2011.1880 PMC3282340 22113029

[eva13633-bib-0019] Coulon, A. (2010). Genhet: An easy‐to‐use R function to estimate individual heterozygosity. Molecular Ecology Resources, 10(1), 167–169. 10.1111/j.1755-0998.2009.02731.x 21565003

[eva13633-bib-0020] Covarrubias, S. , González, C. , & Gutiérrez‐Rodríguez, C. (2021). Effects of natural and anthropogenic features on functional connectivity of anurans: A review of landscape genetics studies in temperate, subtropical and tropical species. Journal of Zoology (1987), 313(3), 159–171. 10.1111/jzo.12851

[eva13633-bib-0021] Cox, K. , Denoël, M. , van Calster, H. , Speybroeck, J. , van de Poel, S. , Lewylle, I. , Verschaeve, L. , van Breusegem, A. , Halfmaerten, D. , Adriaens, D. , & Louette, G. (2021). Scale‐dependent effects of terrestrial habitat on genetic variation in the great crested newt (*Triturus cristatus*). Landscape Ecology, 36(10), 3029–3048. 10.1007/s10980-021-01297-5

[eva13633-bib-0022] Cox, K. , Schepers, R. , Breusegem, A. V. , & Speybroeck, J. (2023). The common ground in landscape effects on gene flow in two newt species in an agroecosystem. Conservation Genetics, 24, 807–826. 10.1007/s10592-023-01539-w

[eva13633-bib-0023] Dervo, B. K. , & Bærum, K. M. (2019). Modellering av bestandsutvikling hos stor‐og småsalamander og frosk. Norsk Institutt for Naturforskning.

[eva13633-bib-0024] Dervo, B. K. , Bærum, K. M. , Skurdal, J. , & Museth, J. (2016). Effects of temperature and precipitation on breeding migrations of amphibian species in southeastern Norway. Scientifica, 2016, 1–8. 10.1155/2016/3174316 PMC4864541 27239371

[eva13633-bib-0025] Dervo, B. K. , Museth, J. , & Skurdal, J. (2018). Assessing the use of artificial hibernacula by the great crested newt (*Triturus cristatus*) and smooth newt (*Lissotriton vulgaris*) in cold climate in Southeast Norway. Diversity (Basel), 10(3), 56. 10.3390/d10030056

[eva13633-bib-0026] Dervo, B. K. , & van der Kooij, J. (2020). Tiltakshåndbok for storsalamander ‐ Erfaringer fra restaurerings‐ og skjøtselstiltak. Norsk Institutt for Naturforskning (NINA).

[eva13633-bib-0027] Dias, P. C. (1996). Sources and sinks in population biology. Trends in Ecology & Evolution, 11(8), 326–330. 10.1016/0169-5347(96)10037-9 21237863

[eva13633-bib-0028] Drake, J. , Lambin, X. , & Sutherland, C. (2022). The value of considering demographic contributions to connectivity: A review. Ecography (Copenhagen), 2022(6), e05552. 10.1111/ecog.05552

[eva13633-bib-0029] Dufresnes, C. , & Perrin, N. (2015). Effect of biogeographic history on population vulnerability in European amphibians. Conservation Biology, 29(4), 1235–1241. 10.1111/cobi.12490 25833793

[eva13633-bib-0030] Epps, C. W. , & Keyghobadi, N. (2015). Landscape genetics in a changing world: Disentangling historical and contemporary influences and inferring change. Molecular Ecology, 24, 6021–6040. 26547281 10.1111/mec.13454

[eva13633-bib-0031] Excoffier, L. , & Lischer, H. E. L. (2010). Arlequin suite ver 3.5: A new series of programs to perform population genetics analyses under Linux and windows. Molecular Ecology Resources, 10, 564–567. 21565059 10.1111/j.1755-0998.2010.02847.x

[eva13633-bib-0032] Farrelly, N. , Ní Dhubháin, Á. , & Nieuwenhuis, M. (2011). Site index of Sitka spruce (Picea sitchensis) in relation to different measures of site quality in Ireland. Revue Canadienne de Recherche forestière, 41(2), 265–278. 10.1139/X10-203

[eva13633-bib-0033] Ficetola, G. F. , & Luigi, M. (2016). Contrasting effects of temperature and precipitation change on amphibian phenology, abundance and performance. Oecologia, 181(3), 683–693. 10.1007/s00442-016-3610-9 27008454

[eva13633-bib-0034] Foll, M. , & Gaggiotti, O. (2008). A genome‐scan method to identify selected loci appropriate for both dominant and codominant markers: A Bayesian perspective. Genetics, 180(2), 977–993. 10.1534/genetics.108.092221 18780740 PMC2567396

[eva13633-bib-0035] Frankham, R. (2015). Genetic rescue of small inbred populations: Meta‐analysis reveals large and consistent benefits of gene flow. Molecular Ecology, 24(11), 2610–2618. 10.1111/mec.13139 25740414

[eva13633-bib-0036] Frankham, R. , Ballou, J. D. , Eldridge, M. D. B. , Lacy, R. C. , Ralls, K. , Dudash, M. R. , & Fenster, C. B. (2011). Predicting the probability of outbreeding depression. Conservation Biology, 25(3), 465–475. 10.1111/j.1523-1739.2011.01662.x 21486369

[eva13633-bib-0037] Frankham, R. , Ballou, J. D. , Ralls, K. , Eldridge, M. , Dudash, M. R. , Fenster, C. B. , Lacy, R. C. , & Sunnucks, P. (2017). Genetic management of fragmented animal and plant populations. Oxford University Press.

[eva13633-bib-0038] Frei, M. , Csencsics, D. , Brodbeck, S. , Schweizer, E. , Bühler, C. , Gugerli, F. , & Bolliger, J. (2016). Combining landscape genetics, radio‐tracking and long‐term monitoring to derive management implications for Natterjack toads (*Epidalea calamita*) in agricultural landscapes. Journal for Nature Conservation, 32, 22–34. 10.1016/j.jnc.2016.04.002

[eva13633-bib-0039] Gauffre, B. , Boissinot, A. , Quiquempois, V. , Leblois, R. , Grillet, P. , Morin, S. , Picard, D. , Ribout, C. , & Lourdais, O. (2022). Agricultural intensification alters marbled newt genetic diversity and gene flow through density and dispersal reduction. Molecular Ecology, 31(1), 119–133. 10.1111/mec.16236 34674328

[eva13633-bib-0040] Goldberg, C. S. , & Waits, L. P. (2010). Comparative landscape genetics of two pond‐breeding amphibian species in a highly modified agricultural landscape. Molecular Ecology, 19(17), 3650–3663. 10.1111/j.1365-294X.2010.04673.x 20723062

[eva13633-bib-0041] Gustafson, D. H. , Andersen, A. S. L. , Mikusiński, G. , & Malmgren, J. C. (2009). Pond quality determinants of occurrence patterns of great crested newts (*Triturus cristatus*). Journal of Herpetology, 43(2), 300–310. 10.1670/07-216R1.1

[eva13633-bib-0042] Haag, C. R. , Riek, M. , Hottinger, J. W. , Pajunen, V. I. , & Ebert, D. (2005). Genetic diversity and genetic differentiation in daphnia Metapopulations with subpopulations of known age. Genetics, 170, 1809–1820. 15937138 10.1534/genetics.104.036814 PMC1449778

[eva13633-bib-0043] Hardy, O. J. , & Vekemans, X. (2002). Spag e d i: A versatile computer program to analyse spatial genetic structure at the individual or population levels. Molecular Ecology Notes, 2(4), 618–620. 10.1046/j.1471-8286.2002.00305.x

[eva13633-bib-0044] Hartel, T. , Nemes, S. , ÖLlerer, K. , CogĂLniceanu, D. A. N. , Moga, C. , & Arntzen, J. W. (2010). Using connectivity metrics and niche modelling to explore the occurrence of the northern crested newt *Triturus cristatus* (Amphibia, Caudata) in a traditionally managed landscape. Environmental Conservation, 37(2), 195–200. 10.1017/S037689291000055X

[eva13633-bib-0045] Hartig, F. , & Lohse, L. (2022). DHARMa: Residual diagnostics for hierarchical (multi‐level/mixed) regression models.

[eva13633-bib-0047] Haugen, H. , Devineau, O. , Heggenes, J. , Østbye, K. , & Linløkken, A. (2022). Predicting habitat properties using remote sensing data: Soil pH and moisture, and ground vegetation cover. Remote Sensing (Basel, Switzerland), 14(20), 5207. 10.3390/rs14205207

[eva13633-bib-0048] Haugen, H. , Linløkken, A. , Østbye, K. , & Heggenes, J. (2020). Landscape genetics of northern crested newt *Triturus cristatus* populations in a contrasting natural and human‐impacted boreal forest. Conservation Genetics, 21, 515–530. 10.1007/s10592-020-01266-6

[eva13633-bib-0049] Hoffmann, A. A. , Sgrò, C. M. , & Kristensen, T. N. (2017). Revisiting adaptive potential, population size, and conservation. Trends in Ecology & Evolution, 32(7), 506–517. 10.1016/j.tree.2017.03.012 28476215

[eva13633-bib-0050] Hofner, B. , Boccuto, L. , & Göker, M. (2015). Controlling false discoveries in high‐dimensional situations: Boosting with stability selection. BMC Bioinformatics, 16(1), 144. 10.1186/s12859-015-0575-3 25943565 PMC4464883

[eva13633-bib-0051] Hofner, B. , Mayr, A. , Robinzonov, N. , & Schmid, M. (2014). Model‐based boosting in R: A hands‐on tutorial using the R package mboost. Computational Statistics, 29(1–2), 3–35. 10.1007/s00180-012-0382-5

[eva13633-bib-0052] Holderegger, R. , & Di Giulio, M. (2010). The genetic effects of roads: A review of empirical evidence. Basic and Applied Ecology, 11(6), 522–531. 10.1016/j.baae.2010.06.006

[eva13633-bib-0053] IUCN . (2009). Triturus cristatus . https://www.iucnredlist.org/species/22212/9365894

[eva13633-bib-0054] Jehle, R. , & Arntzen, J. W. (2000). Post‐breeding migrations of newts (*Triturus cristatus* and *T. marmoratus*) with contrasting ecological requirements. Journal of Zoology, 251(3), 297–306. 10.1111/j.1469-7998.2000.tb01080.x

[eva13633-bib-0055] Johansson, M. , Primmer, C. R. , Sahlsten, J. , & Merilä, J. (2005). The influence of landscape structure on occurrence, abundance and genetic diversity of the common frog, *Rana temporaria* . Global Change Biology, 11(10), 1664–1679. 10.1111/j.1365-2486.2005.1005.x

[eva13633-bib-0056] Kartverket . (2009). Ortofoto Lier bebyggelse 2009 . http://www.norgeibilder.no/?id=490

[eva13633-bib-0057] Kartverket . (2018). Ortofoto Lier Røyen Hurum 2018. http://www.norgeibilder.no/?id=2799

[eva13633-bib-0058] Keller, L. F. , & Waller, D. M. (2002). Inbreeding effects in wild populations. Trends in Ecology & Evolution, 17(5), 230–241. 10.1016/S0169-5347(02)02489-8

[eva13633-bib-0059] Kopelman, N. M. , Mayzel, J. , Jakobsson, M. , Rosenberg, N. A. , & Mayrose, I. (2015). Clumpak: A program for identifying clustering modes and packaging population structure inferences across K. Molecular Ecology Resources, 15(5), 1179–1191. 10.1111/1755-0998.12387 25684545 PMC4534335

[eva13633-bib-0060] Lande, R. (1995). Mutation and conservation. Conservation Biology, 9(4), 782–791. 10.1046/j.1523-1739.1995.09040782.x

[eva13633-bib-0061] Landguth, E. L. , Cushman, S. A. , Schwartz, M. K. , McKelvey, K. S. , Murphy, M. , & Luikart, G. (2010). Quantifying the lag time to detect barriers in landscape genetics. Molecular Ecology, 19(19), 4179–4191. 10.1111/j.1365-294X.2010.04808.x 20819159

[eva13633-bib-0062] Langton, T. E. S. , Beckett, C. L. , & Foster, J. P. (2001). Great crested newt conservation handbook. Froglife.

[eva13633-bib-0063] Lebigre, C. , Turlure, C. , Vandewalle, H. , Binard, F. , Habel, J. C. , & Schtickzelle, N. (2022). Diverging effects of geographic distance and local habitat quality on the genetic characteristics of three butterfly species. Ecological Entomology, 47(5), 842–854. 10.1111/een.13174

[eva13633-bib-0064] Leigh, D. M. , Hendry, A. P. , Vázquez‐Domínguez, E. , & Friesen, V. L. (2019). Estimated six per cent loss of genetic variation in wild populations since the industrial revolution. Evolutionary Applications, 12(8), 1505–1512. 10.1111/eva.12810 31462910 PMC6708419

[eva13633-bib-0065] Li, Y. L. , & Liu, J. X. (2018). StructureSelector: A web‐based software to select and visualize the optimal number of clusters using multiple methods. Molecular Ecology Resources, 18(1), 176–177. 10.1111/1755-0998.12719 28921901

[eva13633-bib-0066] Macbeth, G. M. , Broderick, D. , Buckworth, R. C. , & Ovenden, J. R. (2013). Linkage disequilibrium estimation of effective population size with immigrants from divergent populations: A case study on Spanish mackerel (*Scomberomorus commerson*). G3 (Bethesda), 3(4), 709–717. 10.1534/g3.112.005124 23550119 PMC3618357

[eva13633-bib-0067] Maletzky, A. , Kaiser, R. , & Mikulíček, P. (2010). Conservation genetics of crested newt species *Triturus cristatus* and *T. carnifex* within a contact zone in Central Europe: Impact of interspecific introgression and gene flow. Diversity (Basel), 2(1), 28–46. 10.3390/d2010028

[eva13633-bib-0068] Mangiafic, S. S. (2023). rcompanion: Functions to support extension education program evaluation. https://CRAN.R‐project.org/package=rcompanion

[eva13633-bib-0069] Martínez‐Abraín, A. , & Galán, P. (2018). A test of the substitution–habitat hypothesis in amphibians. Conservation Biology, 32(3), 725–730. 10.1111/cobi.13062 29218741

[eva13633-bib-0070] Martinez‐Abrain, A. , & Jimenez, J. (2016). Anthropogenic areas as incidental substitutes for original habitat. Conservation Biology, 30(3), 593–598. 10.1111/cobi.12644 26483140

[eva13633-bib-0071] McRae, B. H. , Shah, V. B. , & Mohapatra, T. K. (2013). Circuitscape 4 user guide. The Nature Conservancy.

[eva13633-bib-0072] Mills, L. S. , & Allendorf, F. W. (1996). The one‐migrant‐per‐generation rule in conservation and management. Conservation Biology, 10(6), 1509–1518. 10.1046/j.1523-1739.1996.10061509.x

[eva13633-bib-0073] Murphy, M. A. , Dezzani, R. , Pilliod, D. S. , & Storfer, A. (2010). Landscape genetics of high mountain frog metapopulations. Molecular Ecology, 19(17), 3634–3649. 10.1111/j.1365-294X.2010.04723.x 20723055

[eva13633-bib-0074] Newman, R. A. (1998). Ecological constraints on amphibian metamorphosis: Interactions of temperature and larval density with responses to changing food level. Oecologia, 115(1/2), 9–16. 10.1007/s004420050485 28308472

[eva13633-bib-0075] NIBIO . (2019). *AR5 Klassifikasjonssystem: Klassifisering Av Arealressurser* (Vol. 5).

[eva13633-bib-0076] Peakall, R. , & Smouse, P. E. (2012). GenAlEx 6.5: Genetic analysis in excel. Population genetic software for teaching and research—An update. Bioinformatics, 28(19), 2537–2539. 10.1093/bioinformatics/bts460 22820204 PMC3463245

[eva13633-bib-0077] Peterman, W. E. (2018). ResistanceGA: An R package for the optimization of resistance surfaces using genetic algorithms. Methods in Ecology and Evolution, 1638‐1647, 1638–1647. 10.1111/2041-210X.12984

[eva13633-bib-0078] Pritchard, J. K. , Stephens, M. , & Donnelly, P. (2000). Inference of population structure using multilocus genotype data. Genetics, 155(2), 945–959. 10835412 10.1093/genetics/155.2.945 PMC1461096

[eva13633-bib-0079] Pruett, C. L. , & Winker, K. (2008). Effects of sample size on population genetic diversity estimates in song sparrows Melospiza melodia. Journal of Avian Biology, 39(2), 252–256. 10.1111/j.0908-8857.2008.04094.x

[eva13633-bib-0080] Prunier, J. G. , Dubut, V. , Chikhi, L. , Blanchet, S. , & Gaggiotti, O. (2017). Contribution of spatial heterogeneity in effective population sizes to the variance in pairwise measures of genetic differentiation. Methods in Ecology and Evolution, 8(12), 1866–1877. 10.1111/2041-210X.12820

[eva13633-bib-0081] Puechmaille, S. J. (2016). The program structure does not reliably recover the correct population structure when sampling is uneven: Subsampling and new estimators alleviate the problem. Molecular Ecology Resources, 16(3), 608–627. 10.1111/1755-0998.12512 26856252

[eva13633-bib-0082] Rambaut, A. , Drummond, A. J. , Xie, D. , Baele, G. , & Suchard, M. A. (2018). Posterior summarization in Bayesian Phylogenetics using tracer 1.7. Systematic Biology, 67(5), 901–904. 10.1093/sysbio/syy032 29718447 PMC6101584

[eva13633-bib-0083] Rannap, R. , Lohmus, A. , & Briggs, L. (2009). Niche position, but not niche breadth, differs in two coexisting amphibians having contrasting trends in Europe. Diversity and Distributions, 15(4), 692–700. 10.1111/j.1472-4642.2009.00575.x

[eva13633-bib-0084] Rannap, R. , Lõhmus, A. , & Linnamägi, M. (2012). Geographic variation in habitat requirements of two coexisting newt species in Europe. Acta Zoologica Academiae Scientiarum Hungaricae, 58(1), 69–86.

[eva13633-bib-0085] Rivera‐Ortíz, F. A. , Aguilar, R. , Arizmendi, M. D. C. , Quesada, M. , & Oyama, K. (2015). Habitat fragmentation and genetic variability of tetrapod populations. Animal Conservation, 18(3), 249–258. 10.1111/acv.12165

[eva13633-bib-0086] Rothermel, B. B. , & Semlitsch, R. D. (2002). An experimental investigation of landscape resistance of Forest versus old‐field habitats to emigrating juvenile amphibians. Conservation Biology, 16(5), 1324–1332. 10.1046/j.1523-1739.2002.01085.x

[eva13633-bib-0087] Rousset, F. (2008). Genepop’007: A complete re‐implementation of the genepop software for windows and Linux. Molecular Ecology Resources, 8(1), 103–106. 10.1111/j.1471-8286.2007.01931.x 21585727

[eva13633-bib-0088] Rydgren, K. (1993). Herb‐rich spruce forests in W Nordland, N Norway: An ecological and methodological study. Nordic Journal of Botany, 13, 667–690.

[eva13633-bib-0089] Savary, P. , Foltête, J. C. , Moal, H. , Vuidel, G. , Garnier, S. , & Gaggiotti, O. (2021). graph4lg: A package for constructing and analysing graphs for landscape genetics in R. Methods in Ecology and Evolution, 12(3), 539–547. 10.1111/2041-210X.13530

[eva13633-bib-0090] Schmidt, C. , Domaratzki, M. , Kinnunen, R. P. , Bowman, J. , & Garroway, C. J. (2020). Continent‐wide effects of urbanization on bird and mammal genetic diversity. Proceedings of the Royal Society B, 287, 20192497. 32019443 10.1098/rspb.2019.2497 PMC7031673

[eva13633-bib-0091] Shah, R. D. , & Samworth, R. J. (2012). Variable selection with error control: Another look at stability selection. Journal of the Royal Statistical Society, Series B: Statistical Methodology, 75(1), 55–80. 10.1111/j.1467-9868.2011.01034.x

[eva13633-bib-0092] Shemesh, H. , Dener, E. , & Sadeh, A. (2022). Bedrock may dictate the distribution of the fire salamander in the southern border of its global range. Israel Journal of Ecology & Evolution, 1, 1–5. 10.1163/22244662-bja10041

[eva13633-bib-0093] Sol, D. , Lapiedra, O. , & González‐Lagos, C. (2013). Behavioural adjustments for a life in the city. Animal Behaviour, 85(5), 1101–1112. 10.1016/j.anbehav.2013.01.023

[eva13633-bib-0094] SSV . (n.d.). Årsmiddeltemperaturer 1991–2020 . https://kart.vegvesen.no/portal/apps/webappviewer/index.html?id=28a1d1587dec487ba43f1b474f34fa08

[eva13633-bib-0095] Stoutjesdijk, P. , & Barkman, J. J. (2014). Microclimate, vegetation and fauna .

[eva13633-bib-0096] Sztatecsny, M. , Jehle, R. , Schmidt, B. R. , & Arntzen, J. W. (2004). The abundance of premetamorphic newts (*Triturus cristatus*, *T. marmoratus*) as a function of habitat determinants: An a priori model selection approach. Herpetological Journal, 14, 89–97.

[eva13633-bib-0097] Tilghman, J. M. , Ramee, S. W. , & Marsh, D. M. (2012). Meta‐analysis of the effects of canopy removal on terrestrial salamander populations in North America. Biological Conservation, 152, 1–9.

[eva13633-bib-0098] Valdez, J. W. , Gould, J. , & Garnham, J. I. (2021). Global assessment of artificial habitat use by amphibian species. Biological Conservation, 257, 109129. 10.1016/j.biocon.2021.109129

[eva13633-bib-0099] Van Oosterhout, C. , Hutchinson, W. F. , Wills, D. P. M. , & Shipley, P. (2004). Micro ‐ checker: Software for identifying and correcting genotyping errors in microsatellite data. Molecular Ecology Notes, 4(3), 535–538. 10.1111/j.1471-8286.2004.00684.x

[eva13633-bib-0100] Vuorio, V. , Heikkinen, R. K. , & Tikkanen, O.‐P. (2013). Breeding success of the threatened great crested newt in boreal forest ponds. Annales Zoologici Fennici, 50(3), 158–169. 10.5735/086.050.0303

[eva13633-bib-0101] Vuorio, V. , Tikkanen, O.‐P. , Mehtätalo, L. , & Kouki, J. (2015). The effects of forest management on terrestrial habitats of a rare and a common newt species. European Journal of Forest Research, 134(2), 377–388. 10.1007/s10342-014-0858-7

[eva13633-bib-0102] Wang, J. (2017). The computer program structure for assigning individuals to populations: Easy to use but easier to misuse. Molecular Ecology Resources, 17(5), 981–990. 10.1111/1755-0998.12650 28028941

[eva13633-bib-0103] Waples, R. S. , & Do, C. H. I. (2008). Ldne: A program for estimating effective population size from data on linkage disequilibrium. Molecular Ecology Resources, 8(4), 753–756. 10.1111/j.1755-0998.2007.02061.x 21585883

[eva13633-bib-0104] Watts, A. G. , Schlichting, P. E. , Billerman, S. M. , Jesmer, B. R. , Micheletti, S. , Fortin, M.‐J. , Funk, W. C. , Hapeman, P. , Muths, E. , & Murphy, M. A. (2015). How spatio‐temporal habitat connectivity affects amphibian genetic structure. Frontiers in Genetics, 6, 275. 10.3389/fgene.2015.00275 26442094 PMC4561841

[eva13633-bib-0105] Weil, R. R. , Brady, N. C. , & Weil, R. R. (2017). The nature and properties of soils (Fifteenth edition, global edition. ed.). Pearson.

[eva13633-bib-0106] Wielstra, B. , & Arntzen, J. W. (2011). Unraveling the rapid radiation of crested newts (*Triturus cristatus* superspecies) using complete mitogenomic sequences. BMC Evolutionary Biology, 11(1), 162. 10.1186/1471-2148-11-162 21672214 PMC3224112

[eva13633-bib-0107] Wilson, G. A. , & Rannala, B. (2003). Bayesian inference of recent migration rates using multilocus genotypes. Genetics, 163(3), 1177–1191. 12663554 10.1093/genetics/163.3.1177 PMC1462502

