# Supplementary material for: Genetic diversity, gene flow, and landscape resistance in a pond‐breeding amphibian in agricultural and natural forested landscapes in Norway
Source: Evol Appl. 2023 Dec 20;17(1):e13633. doi: 10.1111/eva.13633 (PMC10810167; doi:10.1111/eva.13633)
Supplement: Supplementary file 1 — Tables S1–S5. [file EVA-17-e13633-s002.docx]

**Supplementary Tables**

**Table S1**: Expected heterozygosity (H_E_) and allelic richness (AR) for all populations included in the forested and the agricultural study areas. Allelic richness is calculated using rarefaction with subsampling size = 27.

| **Study area** | **Pop** | **H_E_** | **AR** |
| --- | --- | --- | --- |
| Forested | A | 0.549 | 3.72 |
|  | B | 0.553 | 4.07 |
|  | C | 0.543 | 3.49 |
|  | D | 0.501 | 3.31 |
|  | E | 0.575 | 4.01 |
|  | G | 0.522 | - |
|  | H | 0.509 | 3.61 |
|  | I | 0.429 | 3.14 |
|  | J | 0.544 | 3.43 |
|  | K | 0.547 | 4.24 |
|  | L | 0.568 | 3.9 |
|  | M | 0.539 | 3.3 |
| Agricultural | B-76 | 0.500 | 3.3 |
|  | B-94 | 0.458 | 2.92 |
|  | B-95 | 0.489 | 3.27 |
|  | B-127 | 0.502 | 3.38 |
|  | B-128 | 0.526 | 3.52 |
|  | B-138 | 0.508 | - |
|  | B-139 | 0.519 | 3.16 |
|  | B-141 | 0.427 | 2.97 |
|  | B-143 | 0.352 | 2.61 |
|  | B-148 | 0.419 | 2.66 |
|  | B-149 | 0.410 | 2.56 |
|  | B-150 | 0.319 | - |
|  | B-159 | 0.419 | 2.85 |
|  | B-203 | 0.582 | 3.99 |
|  | B-487 | 0.538 | 3.48 |
|  | B-488 | 0.546 | 3.54 |
|  | B-494 | 0.530 | 3.53 |
|  | B-553 | 0.336 | 2.51 |

**Table S2:** Pairwise Fst for the sampled populations within the forested (left) and the agricultural (right) landscape.

| **Forested** | | |  | **Agricultural** | | |
| --- | --- | --- | --- | --- | --- | --- |
| **Pop** | **Pop** | **Fst** |  | **Pop** | **Pop** | **Fst** |
| A | B | 0.065 |  | B-76 | B-94 | 0.064 |
| A | C | 0.076 |  | B-76 | B-95 | 0.040 |
| A | D | 0.103 |  | B-76 | B-127 | 0.030 |
| A | E | 0.061 |  | B-76 | B-128 | 0.031 |
| A | F | 0.128 |  | B-76 | B-138 | 0.049 |
| A | G | 0.079 |  | B-76 | B-139 | 0.037 |
| A | H | 0.048 |  | B-76 | B-141 | 0.064 |
| A | I | 0.087 |  | B-76 | B-143 | 0.115 |
| A | J | 0.126 |  | B-76 | B-148 | 0.087 |
| A | K | 0.084 |  | B-76 | B-149 | 0.080 |
| A | L | 0.072 |  | B-76 | B-150 | 0.200 |
| A | M | 0.108 |  | B-76 | B-159 | 0.068 |
| B | C | 0.101 |  | B-76 | B-203 | 0.037 |
| B | D | 0.097 |  | B-76 | B-487 | 0.047 |
| B | E | 0.050 |  | B-76 | B-488 | 0.038 |
| B | F | 0.080 |  | B-76 | B-494 | 0.037 |
| B | G | 0.059 |  | B-76 | B-553 | 0.121 |
| B | H | 0.055 |  | B-94 | B-95 | 0.018 |
| B | I | 0.115 |  | B-94 | B-127 | 0.090 |
| B | J | 0.126 |  | B-94 | B-128 | 0.085 |
| B | K | 0.094 |  | B-94 | B-138 | 0.083 |
| B | L | 0.078 |  | B-94 | B-139 | 0.074 |
| B | M | 0.105 |  | B-94 | B-141 | 0.072 |
| C | D | 0.128 |  | B-94 | B-143 | 0.140 |
| C | E | 0.092 |  | B-94 | B-148 | 0.129 |
| C | F | 0.129 |  | B-94 | B-149 | 0.127 |
| C | G | 0.115 |  | B-94 | B-150 | 0.249 |
| C | H | 0.110 |  | B-94 | B-159 | 0.095 |
| C | I | 0.125 |  | B-94 | B-203 | 0.066 |
| C | J | 0.130 |  | B-94 | B-487 | 0.070 |
| C | K | 0.082 |  | B-94 | B-488 | 0.066 |
| C | L | 0.084 |  | B-94 | B-494 | 0.070 |
| C | M | 0.098 |  | B-94 | B-553 | 0.164 |
| D | E | 0.062 |  | B-95 | B-127 | 0.064 |
| D | F | 0.168 |  | B-95 | B-128 | 0.056 |
| D | G | 0.120 |  | B-95 | B-138 | 0.069 |
| D | H | 0.085 |  | B-95 | B-139 | 0.054 |
| D | I | 0.122 |  | B-95 | B-141 | 0.055 |
| D | J | 0.154 |  | B-95 | B-143 | 0.122 |
| D | K | 0.112 |  | B-95 | B-148 | 0.107 |
| D | L | 0.107 |  | B-95 | B-149 | 0.108 |
| D | M | 0.134 |  | B-95 | B-150 | 0.211 |
| E | F | 0.113 |  | B-95 | B-159 | 0.073 |
| E | G | 0.095 |  | B-95 | B-203 | 0.049 |
| E | H | 0.041 |  | B-95 | B-487 | 0.061 |
| E | I | 0.113 |  | B-95 | B-488 | 0.052 |
| E | J | 0.120 |  | B-95 | B-494 | 0.054 |
| E | K | 0.065 |  | B-95 | B-553 | 0.140 |
| E | L | 0.089 |  | B-127 | B-128 | 0.009 |
| E | M | 0.092 |  | B-127 | B-138 | 0.062 |
| F | G | 0.153 |  | B-127 | B-139 | 0.046 |
| F | H | 0.145 |  | B-127 | B-141 | 0.063 |
| F | I | 0.191 |  | B-127 | B-143 | 0.102 |
| F | J | 0.158 |  | B-127 | B-148 | 0.088 |
| F | K | 0.113 |  | B-127 | B-149 | 0.087 |
| F | L | 0.127 |  | B-127 | B-150 | 0.179 |
| F | M | 0.184 |  | B-127 | B-159 | 0.067 |
| G | H | 0.058 |  | B-127 | B-203 | 0.021 |
| G | I | 0.123 |  | B-127 | B-487 | 0.027 |
| G | J | 0.145 |  | B-127 | B-488 | 0.025 |
| G | K | 0.109 |  | B-127 | B-494 | 0.023 |
| G | L | 0.066 |  | B-127 | B-553 | 0.121 |
| G | M | 0.092 |  | B-128 | B-138 | 0.065 |
| H | I | 0.093 |  | B-128 | B-139 | 0.051 |
| H | J | 0.141 |  | B-128 | B-141 | 0.057 |
| H | K | 0.090 |  | B-128 | B-143 | 0.093 |
| H | L | 0.080 |  | B-128 | B-148 | 0.080 |
| H | M | 0.092 |  | B-128 | B-149 | 0.080 |
| I | J | 0.177 |  | B-128 | B-150 | 0.162 |
| I | K | 0.103 |  | B-128 | B-159 | 0.067 |
| I | L | 0.083 |  | B-128 | B-203 | 0.020 |
| I | M | 0.102 |  | B-128 | B-487 | 0.033 |
| J | K | 0.098 |  | B-128 | B-488 | 0.023 |
| J | L | 0.078 |  | B-128 | B-494 | 0.018 |
| J | M | 0.169 |  | B-128 | B-553 | 0.106 |
| K | L | 0.062 |  | B-138 | B-139 | 0.034 |
| K | M | 0.101 |  | B-138 | B-141 | 0.121 |
| L | M | 0.099 |  | B-138 | B-143 | 0.173 |
|  |  |  |  | B-138 | B-148 | 0.144 |
|  |  |  |  | B-138 | B-149 | 0.144 |
|  |  |  |  | B-138 | B-150 | 0.261 |
|  |  |  |  | B-138 | B-159 | 0.127 |
|  |  |  |  | B-138 | B-203 | 0.057 |
|  |  |  |  | B-138 | B-487 | 0.066 |
|  |  |  |  | B-138 | B-488 | 0.054 |
|  |  |  |  | B-138 | B-494 | 0.054 |
|  |  |  |  | B-138 | B-553 | 0.188 |
|  |  |  |  | B-139 | B-141 | 0.074 |
|  |  |  |  | B-139 | B-143 | 0.135 |
|  |  |  |  | B-139 | B-148 | 0.122 |
|  |  |  |  | B-139 | B-149 | 0.116 |
|  |  |  |  | B-139 | B-150 | 0.248 |
|  |  |  |  | B-139 | B-159 | 0.086 |
|  |  |  |  | B-139 | B-203 | 0.043 |
|  |  |  |  | B-139 | B-487 | 0.052 |
|  |  |  |  | B-139 | B-488 | 0.046 |
|  |  |  |  | B-139 | B-494 | 0.046 |
|  |  |  |  | B-139 | B-553 | 0.157 |
|  |  |  |  | B-141 | B-143 | 0.054 |
|  |  |  |  | B-141 | B-148 | 0.061 |
|  |  |  |  | B-141 | B-149 | 0.058 |
|  |  |  |  | B-141 | B-150 | 0.205 |
|  |  |  |  | B-141 | B-159 | 0.039 |
|  |  |  |  | B-141 | B-203 | 0.055 |
|  |  |  |  | B-141 | B-487 | 0.063 |
|  |  |  |  | B-141 | B-488 | 0.069 |
|  |  |  |  | B-141 | B-494 | 0.060 |
|  |  |  |  | B-141 | B-553 | 0.074 |
|  |  |  |  | B-143 | B-148 | 0.052 |
|  |  |  |  | B-143 | B-149 | 0.039 |
|  |  |  |  | B-143 | B-150 | 0.184 |
|  |  |  |  | B-143 | B-159 | 0.070 |
|  |  |  |  | B-143 | B-203 | 0.087 |
|  |  |  |  | B-143 | B-487 | 0.102 |
|  |  |  |  | B-143 | B-488 | 0.115 |
|  |  |  |  | B-143 | B-494 | 0.107 |
|  |  |  |  | B-143 | B-553 | 0.045 |
|  |  |  |  | B-148 | B-149 | 0.014 |
|  |  |  |  | B-148 | B-150 | 0.108 |
|  |  |  |  | B-148 | B-159 | 0.040 |
|  |  |  |  | B-148 | B-203 | 0.078 |
|  |  |  |  | B-148 | B-487 | 0.095 |
|  |  |  |  | B-148 | B-488 | 0.101 |
|  |  |  |  | B-148 | B-494 | 0.092 |
|  |  |  |  | B-148 | B-553 | 0.028 |
|  |  |  |  | B-149 | B-150 | 0.130 |
|  |  |  |  | B-149 | B-159 | 0.041 |
|  |  |  |  | B-149 | B-203 | 0.075 |
|  |  |  |  | B-149 | B-487 | 0.084 |
|  |  |  |  | B-149 | B-488 | 0.098 |
|  |  |  |  | B-149 | B-494 | 0.086 |
|  |  |  |  | B-149 | B-553 | 0.023 |
|  |  |  |  | B-150 | B-159 | 0.177 |
|  |  |  |  | B-150 | B-203 | 0.162 |
|  |  |  |  | B-150 | B-487 | 0.198 |
|  |  |  |  | B-150 | B-488 | 0.185 |
|  |  |  |  | B-150 | B-494 | 0.197 |
|  |  |  |  | B-150 | B-553 | 0.142 |
|  |  |  |  | B-159 | B-203 | 0.069 |
|  |  |  |  | B-159 | B-487 | 0.072 |
|  |  |  |  | B-159 | B-488 | 0.085 |
|  |  |  |  | B-159 | B-494 | 0.069 |
|  |  |  |  | B-159 | B-553 | 0.065 |
|  |  |  |  | B-203 | B-487 | 0.026 |
|  |  |  |  | B-203 | B-488 | 0.024 |
|  |  |  |  | B-203 | B-494 | 0.023 |
|  |  |  |  | B-203 | B-553 | 0.101 |
|  |  |  |  | B-487 | B-488 | 0.017 |
|  |  |  |  | B-487 | B-494 | 0.022 |
|  |  |  |  | B-487 | B-553 | 0.124 |
|  |  |  |  | B-488 | B-494 | 0.020 |
|  |  |  |  | B-488 | B-553 | 0.128 |
|  |  |  |  | B-494 | B-553 | 0.110 |

**Table S3:** Marginal R^2^ for single predictor resistance surfaces optimized separately for the forested landscape (left) and the agricultural landscape (right), using Dps as response and two runs.

| **Surface** | **R^2^m** | |  | **Surface** | **R^2^m** | |
| --- | --- | --- | --- | --- | --- | --- |
|  | Run 1 | Run 2 |  |  | Run 1 | Run 2 |
| Moisture 2008-09 | 0.35 | 0.31 |  | Moisture 2008-09 | 0.65 | 0.65 |
| Moisture, no canopy | 0.30 | 0.29 |  | Landcover2009 | 0.64 | 0.63 |
| Moisture 2017 | 0.24 | 0.26 |  | Landcover2017 | 0.64 | 0.63 |
| Field vegetation 2017 | 0.25 | 0.23 |  | Soil pH | 0.63 | 0.61 |
| Soil pH | 0.20 | 0.15 |  | Moisture, no canopy | 0.62 | 0.62 |
| Field vegetation 2008-09 | 0.19 | 0.19 |  | Field vegetation 2009 | 0.61 | 0.61 |
| Landcover | 0.16 | 0.16 |  | Moisture 2017 | 0.60 | 0.60 |
| Distance | 0.10 | 0.10 |  | Field vegetation2017 | 0.59 | 0.58 |
|  |  |  |  | Distance | 0.59 | 0.58 |

**Table S4:** Results from the bootstrap analysis of the landscape resistance models for the forested landscape*.* Models are ranked according to the average AICc. In addition, average marginal R^2^ and the percentage of bootstrap samples where each model was ranked as the best model (% top) using AICc.

| **Rank** | **Model** | **avg. AICc** | **avg. R^2^m** | **% top** |
| --- | --- | --- | --- | --- |
| 1 | Moisture + Land cover | −115.99 | 0.48 | 26.4 |
| 2 | Moisture | −115.93 | 0.31 | 26.5 |
| 3 | Moisture + Soil pH | −115.73 | 0.46 | 22.7 |
| 4 | Moisture | −115.67 | 0.30 | 4.7 |
| 5 | Moisture + Land cover | −115.29 | 0.46 | 0.0 |
| 6 | Land cover | −114.23 | 0.18 | 19.3 |
| 7 | Field vegetation | −113.77 | 0.22 | 0.0 |
| 8 | Field vegetation | −113.66 | 0.24 | 0.0 |
| 9 | Soil pH | −113.14 | 0.15 | 0.0 |
| 10 | Soil pH | −113.07 | 0.20 | 0.0 |
| 11 | Land cover | −112.40 | 0.14 | 0.4 |
| 12 | Distance | −112.07 | 0.10 | 0.0 |
| 13 | Distance | −112.07 | 0.10 | 0.0 |
| 14 | Moisture + Field vegetation | −112.05 | 0.34 | 0.0 |
| 15 | Moisture + Field vegetation | −111.82 | 0.33 | 0.0 |
| 16 | Moisture + Soil pH | −111.02 | 0.24 | 0.0 |
| 17 | Field vegetation + Land cover | −110.07 | 0.20 | 0.0 |
| 18 | Field vegetation + Land cover | −110.04 | 0.20 | 0.0 |
| 19 | Soil pH + Field vegetation | −109.63 | 0.22 | 0.0 |
| 20 | Soil pH + Field vegetation | −109.63 | 0.22 | 0.0 |
| 21 | Land cover + Soil pH | −109.32 | 0.18 | 0.0 |
| 22 | Land cover + Soil pH | −109.28 | 0.17 | 0.0 |

**Table S5:** Results from the bootstrap analysis of the landscape resistance models for the agricultural landscape*.* Models are ranked according to the average AICc. In addition, average marginal R^2^ and the percentage of bootstrap samples where each model was ranked as the best model (% top) using AICc.

| **Rank** | **Model** | **avg. AICc** | **avg. R^2^m** | **% top** |
| --- | --- | --- | --- | --- |
| 1 | Land cover + Soil pH | −266.73 | 0.70 | 39.8 |
| 2 | Land cover + Soil pH | −266.20 | 0.69 | 5.3 |
| 3 | Soil pH | −263.89 | 0.63 | 28.9 |
| 4 | Moisture | −263.65 | 0.65 | 9.6 |
| 5 | Moisture | −263.52 | 0.65 | 2.9 |
| 6 | Soil pH + Field vegetation | −261.05 | 0.69 | 4.6 |
| 7 | Soil pH + Field vegetation | −260.98 | 0.69 | 1.2 |
| 8 | Moisture + Soil pH | −259.69 | 0.65 | 5.1 |
| 9 | Moisture + Soil pH | −258.39 | 0.61 | 0.0 |
| 10 | Moisture + Land cover | −258.03 | 0.64 | 0.3 |
| 11 | Moisture + Land cover | −258.03 | 0.65 | 0.2 |
| 12 | Soil pH | −257.56 | 0.61 | 0.0 |
| 13 | Land cover | −255.22 | 0.63 | 0.6 |
| 14 | Moisture + Field vegetation | −254.25 | 0.66 | 0.0 |
| 15 | Moisture + Field vegetation | −254.09 | 0.67 | 1.2 |
| 16 | Field vegetation + Land cover | −253.11 | 0.64 | 0.3 |
| 17 | Field vegetation + Land cover | −252.46 | 0.64 | 0.0 |
| 18 | Field vegetation | −252.44 | 0.62 | 0.0 |
| 19 | Distance | −251.99 | 0.58 | 0.2 |
| 20 | Distance | −251.80 | 0.58 | 0.0 |
| 21 | Field vegetation | −250.85 | 0.60 | 0.0 |
| 22 | Land cover | −248.07 | 0.61 | 0.0 |
