# Supplementary material for: Genetic diversity, gene flow, and landscape resistance in a pond‐breeding amphibian in agricultural and natural forested landscapes in Norway
Source: Evol Appl. 2023 Dec 20;17(1):e13633. doi: 10.1111/eva.13633 (PMC10810167; doi:10.1111/eva.13633)
Supplement: Supplementary file 2 — Text S1. [file EVA-17-e13633-s001.docx]

**Supplementary text (material and methods)**

**Environmental variables**

Soil pH, moisture, and field vegetation cover were modeled from LiDAR data and natural resource maps in a previous study, for the study areas in Notodden and Lier (Haugen et al 2022). These models were used with some adaptions.

The original soil pH model did not include the effect of bedrock calcium content because the variation in bedrock types was not sufficiently sampled. Since the models were based on vegetation types as bio-indicators for pH and moisture, we could collect additional samples from existing vegetation maps. We collected 44 new data points from Lier and together with the previous 56 vegetation samples, rerun the whole analysis. The final model included sediment type, site index, bedrock calcium content, and topographical wetness. The regression equation was used to create the soil pH map using the raster calculator in ArcGIS.

In the original soil moisture model (Haugen et al 2022), dry and very wet vegetation types were less represented by the samples. Therefore, eight more samples were collected in the driest areas in Notodden, using a handheld GPS. The random forest classifier in ArcMap was used to model soil moisture with the predictors from the original model (Haugen et al 2022): sediment type, site index, solar radiation load and topographic position, with an additional new predictor representing soil depth. The predictor was derived from an existing natural resource map (NIBIO,2019), here soil depth was classified on an ordinal scale (3 levels). To better represent very wet areas, we estimated dept-to-water- table (DTW) and included areas where the water table was estimated at a depth of 1m or less. DTW was calculated using a 1m resolution terrain model and an initiation threshold of 1ha. A total of three soil moisture models were created using three different versions of solar radiation load: 1. Solar radiation load including canopy from the year 2017, 2. Solar radiation load including canopy from 2008-09 and 3. Solar radiation load excluding the effect of canopy.

The field vegetation cover model in Haugen et al (2022) consists of a LiDAR-based model of below-canopy-sunlight, forest type (spruce-dominated, pine-dominated, and deciduous forest), and the interaction between these two predictors. The original model was based on the state of the canopy cover in the year 2017, thus we included an additional model where light conditions were estimated from the canopy cover of the period 2008-09.

To approximate the amount of sunlight hitting the pond surfaces, we used the solar radiation variable from the soil moisture model from 2017 to estimate the median amount of sunlight per square meter.
